# The Mediterranean-Dietary Approaches to Stop Hypertension Intervention for Neurodegenerative Delay (MIND) Diet for the Aging Brain: A Systematic Review

**DOI:** 10.1016/j.advnut.2024.100184

**Published:** 2024-02-03

**Authors:** Annick PM van Soest, Sonja Beers, Ondine van de Rest, Lisette CPGM de Groot

**Affiliations:** Division of Human Nutrition and Health, Wageningen University & Research, Wageningen, The Netherlands

**Keywords:** MIND diet, dietary pattern, nutrition, diet, cognitive function, Alzheimer’s disease, healthy aging, older adults, elderly

## Abstract

The Mediterranean-Dietary Approaches to Stop Hypertension Intervention for Neurodegenerative Delay (MIND) diet seems a promising approach to preserve brain function during aging. Previous systematic reviews have demonstrated benefits of the MIND diet for cognition and dementia, though an update is needed. Additionally, other outcomes relevant to brain aging have not been summarized. Therefore, this systematic review aims to give an up-to-date and complete overview on human studies that examined the MIND diet in relation to brain aging outcomes in adults aged ≥40 y. Ovid Medline, Web of Science core collection, and Scopus were searched up to July 25, 2023. Study quality was assessed using the Newcastle–Ottawa Scale and the Cochrane Risk-of-Bias tool. We included 40 articles, of which 32 were unique cohorts. Higher MIND diet adherence was protective of dementia in 7 of 10 cohorts. Additionally, positive associations were demonstrated in 3 of 4 cohorts for global cognition and 4 of 6 cohorts for episodic memory. The protective effects of the MIND diet on cognitive decline are less apparent, with only 2 of 7 longitudinal cohorts demonstrating positive associations for global decline and 1 of 6 for episodic memory decline. For other brain outcomes (domain-specific cognition, cognitive impairments, Parkinson’s disease, brain volume, and pathology), results were mixed or only few studies had been performed. Many of the cohorts demonstrating protective associations were of North American origin, raising the question if the most favorable diet for healthy brain aging is population-dependent. In conclusion, this systematic review provides observational evidence for protective associations between the MIND diet and global cognition and dementia risk, but evidence for other brain outcomes remains mixed and/or limited. The MIND diet may be the preferred diet for healthy brain aging in North American populations, though evidence for other populations seems less conclusive.

This review was registered at PROSPERO as CRD42022254625.

**Statement of Significance**

In the field of nutrition and brain aging research, dietary patterns, particularly the MIND diet, are a prominent area of interest. This systematic review provides an overview of studies examining the MIND diet in relation to brain aging. It updates previous systematic reviews with respect to cognition, cognitive decline, and dementia and extends to other brain aging outcomes including Parkinson’s disease, brain volume, and pathology.

## Introduction

With increasing age, the functioning of the brain gradually declines. Processing speed, executive function, and episodic memory performance start to become impaired during midlife and further decline into older age [1]. This decline in cognitive performance is accompanied by changes in the brain. For example, the volume of the brain shrinks, and abnormal proteins accumulate. In case of accelerated aging, these and other changes may eventually lead to age-related brain diseases, including various types of dementia and Parkinson’s disease (PD) [2].

As it is not possible to completely stop brain aging or cure age-related brain diseases, there is increasing interest in preventive strategies to ensure optimal brain aging. Nutrition is considered an important lifestyle factor that can influence the brain aging trajectory. Over recent decades, the research field has shifted from studying single nutrients and foods toward dietary patterns [3]. Studying dietary patterns is thought to be a more powerful approach to unravel the role of nutrition in brain aging, as it allows to capture synergistic beneficial effects of nutrients. Indeed, evidence for dietary patterns is stronger than that for single nutrients and foods [3].

A dietary pattern that seems promising is the Mediterranean-Dietary Approaches to Stop Hypertension (DASH) Intervention for Neurodegenerative Delay (MIND) diet, which is specifically developed to preserve brain function during aging. The MIND diet is a hybrid of the Mediterranean and DASH diets and further emphasizes intake of food groups with neuroprotective properties, including berries and leafy green vegetables. According to the developers of the MIND diet, it is more protective against cognitive decline [4] and Alzheimer’s disease (AD) [5] than the Mediterranean and DASH diets.

The possible beneficial role of the MIND diet in healthy brain aging has been summarized systematically in 5 reviews and 2 meta-analyses [[6], [7], [8], [9], [10], [11], [12]]. However, these previously published articles are either in need of an update and/or only focused on cognitive functioning and/or dementia rather than taking a broader perspective on the aging brain. To this end, we aim *1*) to give an updated overview on the MIND diet in relation to cognitive functioning, cognitive decline, and dementia risk and *2*) to extend this overview to other brain aging outcomes, including neuroimaging and pathology outcomes and incidence of other age-related neurodegenerative diseases.

## Methods

### Protocol registration

We conducted this systematic review in accordance with the PRISMA guidelines [13]. The study protocol was registered in PROSPERO (CRD42022254625).

### Information sources and search strategy

A systematic search was performed in 3 databases: Ovid Medline, Web of Science core collection, and Scopus. No date restrictions were applied. An initial search was conducted on October 12, 2022. After this date, an automatic alert was set up within these databases to identify new articles published until July 25, 2023. The searches were conducted using predefined terms related to the MIND diet and the aging brain (full search strategy in Supplemental Table 1A–C). Search terms were determined in consultation with a librarian.

### Study selection and eligibility criteria

The web tool CADIMA was used to organize the systematic review [14]. Duplicates were automatically removed by the web tool.

Two researchers (AvS and SB) independently reviewed the title and abstract of all obtained literature and subsequently the full text for eligibility. For eligibility, the following criteria were applied: *1*) The study was a human observational or interventional study. Meta-analyses, reviews, commentaries, editorials, abstracts, unpublished studies, letters, news, or newspaper articles were excluded; *2*) The study population comprised middle-aged and older individuals, all aged ≥40 y. If only mean age was stated, the mean age minus 2 times the standard deviation had to be ≥40 y. This age cutoff was chosen because brain aging is already present during midlife [15]; *3*) The exposure variable was a measure of MIND diet adherence (observational studies) or a MIND diet intervention (interventional studies); *4*) The comparator was lower adherence to the MIND diet (observational studies) or no MIND diet intervention (interventional studies); *5*) The outcome measure was related to brain aging, including cognitive performance, cognitive decline, incidence of any type of dementia or PD, or brain volume and pathology outcomes. Outcome measures related to depression, brain tumors, and/or multiple sclerosis were excluded; *6*) An effect size was given for the association between MIND diet exposure and brain aging outcome; and *7*) The article was published in English in a peer-reviewed journal. The 2 researchers (AvS and SB) resolved disagreements by discussion. Any remaining disagreements were discussed among all contributing authors until consensus was reached.

### Data extraction

Data extraction was independently performed by 2 researchers (AvS and SB). The following variables were extracted from eligible studies: first author, year of publication, country, name of study, study design, study duration (duration of follow-up or intervention), sample size, description of the study population, description of the exposure variable, outcome measure(s), results including effect size, and covariates. In case various models were analyzed with different covariates, we collected the results of the most extensively adjusted model. Studies were organized based on outcome variable, with the exception of the randomized controlled trials (RCTs), which were tabled together. Outcome variables were categorized as cognitive function, cognitive decline, dementia, cognitive impairments, PD, and brain volume and pathology.

### Quality assessment

Two independent researchers (AvS and SB) assessed the quality of the included studies. The instruments used for quality assessment were based on the Cochrane Handbook for Systematic Reviews of Interventions [16]. The Newcastle–Ottawa Scale (NOS) was used to rate the quality of observational cohort and case–control studies (Supplemental Tables 2 and 3) and an adapted version of the NOS for quality of cross-sectional studies [17,18] (Supplemental Table 4). Cohort and case–control studies were scored on the domains “selection,” “comparability,” and “outcome/exposure,” with maximum scores for the individual domains being 4, 2, and 4, respectively. The maximum score for cross-sectional studies was 7, of which a maximum of 3, 2, and 2 points could be retrieved from the domains selection, comparability, and outcome, respectively. Quality was categorized as either good, fair, or poor. Threshold scores for categorizing the study quality are shown in Supplemental Tables 2–4.

In addition, the risk of bias of RCTs was assessed using the Cochrane Risk-of-Bias tool for randomized trials (ROB2) [19]. ROB2 is structured into 5 domains of bias: randomization process, deviations from intended interventions, missing outcomes, measurement of the outcome, and selection of reported result. Within each domain, a series of signaling questions can be answered as “yes,” “no,” “do not know or unclear,” or “not applicable.” These answers lead to the judgment of “low risk of bias,” “some concerns,” or “high risk of bias.”

Disagreements were resolved by discussion between 2 researchers (AvS and SB). Remaining disagreements were discussed among all contributing authors until consensus was reached.

## Results

### Identification and selection

**FIGURE 1:**
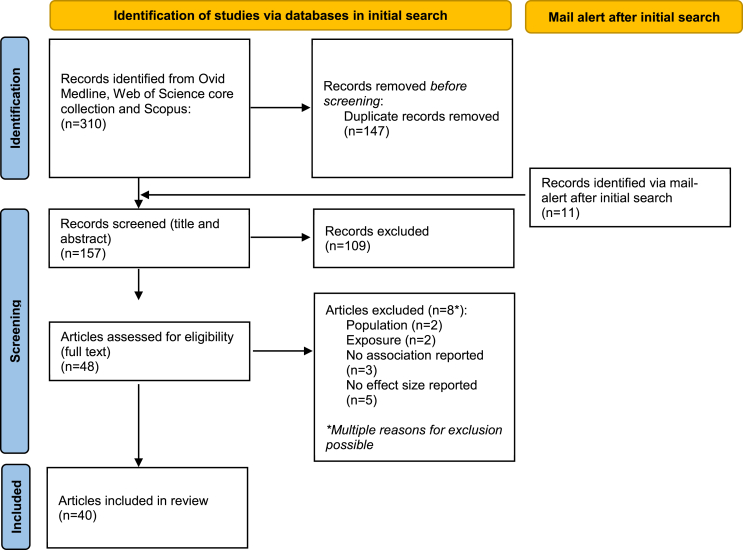
PRISMA flow chart summarizing literature search, study identification, and selection.

Out of the 321 studies identified in the database searches, a total of 40 articles met the inclusion criteria (Figure 1).

### Study characteristics

The characteristics of the 40 articles are presented in TABLE 1, TABLE 2, TABLE 3, TABLE 4, TABLE 5, TABLE 6, TABLE 7 [4,5,11,12,[20], [21], [22], [23], [24], [25], [26], [27], [28], [29], [30], [31], [32], [33], [34], [35], [36], [37], [38], [39], [40], [41], [42], [43], [44], [45], [46], [47], [48], [49], [50], [51], [52], [53], [54], [55]], and quality assessments are presented in Supplemental Tables 5–8. Two of the included articles were RCTs, and 38 articles had an observational design. Among the included articles, some cohorts were used multiple times. The Rush Memory and Aging Project (MAP) (*n* = 8) [4,5,[20], [21], [22], [23], [24], [25]], Health and Retirement Study (*n* = 3) [11,26,27], Framingham Heart Study (*n* = 2) [11,28], United Kingdom Biobank (*n* = 2) [29,30], and the Women’s Health Initiative (*n* = 2) [20,31] cohorts were used by multiple articles. This resulted in the inclusion of 32 unique cohorts in this systematic review.

**TABLE 1:** Description of included cross-sectional studies describing the association between MIND diet and cognitive function

Author (year)	Study (country)	Sample size (n)	Population	Exposure	Outcome	Results	Covariates	Study quality^1^
McEvoy (2017) [26]	Health and Retirement Study (USA)	5907	Older adults (mean age 67.8 ± 10.8y), without history of stroke or dementia	15-MIND diet adherence (tertiles) based on 163-item sFFQ	Cognition measured by (1) Global cognition (range 0–27, based on immediate, delayed word list, backward counting, and serial 7 subtraction) (2) Impaired cognition (defined by more than 1SD (=4.3 points) below mean global cognition score)	(1) Mean (SE) for global cognition T1: 14.9 (0.10) T2: 15.2 (0.09) T3: 15.6 (0.09) p for trend: **<0.001** (2) OR (95% CI) for impaired cognition T1 vs T2: 0.85 (0.70, 1.03), p = 0.10 T1 vs T3: **0.70 (0.56, 0.86), p** = **0.001**	Sex, age, race, low education attainment, current smoking, total wealth, obesity, hypertension, diabetes mellitus, physical inactivity, depression, total energy intake	Good
Ahn (2022) [27]	Health and Retirement Study (USA)	3463	Older adults (≥50y), without history of stroke or dementia	15-MIND diet adherers (score ≥7.5) vs non-adherers (<7.5), based on 163-item sFFQ	Cognition measured by (1) Global cognition (range 0–27, based on immediate and delayed recall word list, serial seven subtraction, backward counting); (2) Impaired cognition (defined by more than 1SD (=4.5 points) below mean global cognition score)	(1) Mean difference (95% CI) in global cognition In physically inactive individuals: **0.81 (0.50, 1.11), p**<**0.001** In regular physically active individuals: **0.60 (0.08, 1.12), p<0.001** (2) OR (95% CI) of impaired cognition In physically inactive individuals **0.68 (0.54, 0.86), p**<**0.01**; In regular physically active individuals 0.73 (0.48, 1.11), p > 0.05	Age, sex, race, education, annual income, smoking history, hypertension, diabetes mellitus, depression, obesity	Good
Van Lent (2021) [28]	Framingham Heart Study, offspring cohort (USA)	2092	Older adults (mean age 61 ± 9y), free of dementia	15-MIND diet adherence (continuous) based on 126-item sFFQ	Cognition measured by: (1) Visual Reproductions Delayed Recall; (2) Logical Memory Delayed Recall; (3) Trail Making Test (TMT) A; (4) Trail Making Test B/A; (5) Hooper Visual Organization Test; (6) Similarities; (7) Global cognition (comp. of tests above)	β±SE (1) Visual reproductions delayed recall: **0.03** ± **0.01, p** = **0.01** (2) Logical memory delayed recall: **0.03** ± **0.01, p** = **0.02** (3) TMT A: **0.03** ± **0.01, p** = **0.01** (4) TMT B/A: 0.01 ± 0.01, p = 0.30 (5) Hooper visual organization test: 0.01 ± 0.01, p = 0.28 (6) Similarities: **0.03** ± **0.01, p** = **0.02** (7) Global cognition: **0.03** ± **0.01, p** = **0.004**	Age, age squared, sex, ApoE4 status, total energy intake, education, BMI, physical activity, smoking, diabetes, CVD, depressive symptoms, anti-hypertensive medication, systolic blood pressure, total cholesterol to HDL ratio, time interval between FFQ and outcome measure.	Good
Berendsen (2018) [39]	Nurses' Health Study (USA)	16058	Older women (≥70y), free of stroke, free of dementia	15-MIND diet adherence (quantiles) based on 116-item sFFQ	Cognition measured by (1) TICS; (2) Verbal (episodic) memory (comp. of immediate and delayed recalls of the East Boston Memory Test and delayed recall of the TICS); (3) Global cognition (comp. of aforementioned tests and category fluency digit span backward)	Mean difference (95% CI) (1) TICS: Q1 vs Q2: 0.05 (−0.18, 0.07) Q1 vs Q3: 0.00 (−0.13, 0.12) Q1 vs Q4: −0.02 (−0.14, 0.11) Q1 vs Q5: 0.09 (−0.21, 0.04) (2) Verbal memory: Q1 vs Q2: 0.01 (−0.02, 0.05) Q1 vs Q3: 0.02 (−0.01, 0.05) Q1 vs Q4: 0.02 (−0.01, 0.06) Q1 vs Q5: **0.04 (0.01, 0.07)** (3) Global cognition: Q1 vs Q2: 0.00 (−0.02, 0.03) Q1 vs Q3: 0.00 (−0.03, 0.03) Q1 vs Q4: 0.00 (−0.03, 0.03) Q1 vs Q5: 0.00 (−0.03, 0.03)	Age, education, physical activity, calorie intake, alcohol intake, smoking status, multivitamin use, BMI, depression, history of high blood pressure, hypercholesterolemia, myocardial infarction, diabetes mellitus.	Fair
Boumenna (2022) [40]	The Boston Puerto Rican Health Study (USA)	1081	Middle-aged to older adults (mean age 52.7 ± 7.9)	15-MIND diet adherence (quantiles and continuous) assessed by FFQ	Global cognition (comp. of MMSE, 16 word list learning, digit span forward and backward, stroop test, clock drawing and figure copying, verbal fluency)	β (95% CI) Q1 vs Q2: −0.065 (−0.162, 0.033) Q1 vs Q3: −0.005 (−0.085, 0.075) Q1 vs Q4: 0.047 (−0.035, 0.129) Q1 vs Q5: **0.092 (0.002, 0.182)** Continuous: **0.027 (0.008, 0.046), p** = **0.0062**	Age, sex, BMI, physical activity score, diabetes, hypertension, educational level, smoking, alcohol use, APOE4, energy intake, job complexity score, poverty index	Good
Huang (2023) [12]	China Health and Nutrition Survey (China)	4066	Older adults (≥55y), free of dementia	12-MIND diet adherence (tertiles and continuous) based on 3 24 h dietary recalls	Cognition measured by (1) Global cognition, comp. of items of TICS-m; (2) Verbal memory, comp. of immediate and delayed recall	β (95% CI) (1) Global cognition/TICS-m T1 vs T2: 0.017 (−0.027, 0.061) T1 vs T3: **0.071 (0.026, 0.116)** Continuous (per 3 points): **0.110 (0.060, 0.159)** (2) Verbal memory T1 vs T2: 0.003 (−0.042, 0.049) T1 vs T3: **0.068 (0.021, 0.115)** Continuous (per 3 points): **0.102 (0.051, 0.153)**	Age, age square, sex, education, residence, region, income, smoking status, drinking status, BMI, total energy, physical activities, hypertension, diabetes, myocardial infarction	Good
Wesselman (2021) [41]	The DZNE-Longitudinal Cognitive Impairment and Dementia Study (Germany)	383	Older adults (mean age 69.3 ± 5.6y), free of dementia	15-MIND diet adherence (continuous) based on 148-item sFFQ	Cognition measured by (1) Memory (comp. of cognitive subscale word list, delayed recall and cognition, free and cued selective reminding test, free recall and cue efficiency, Wechsler Memory Scale, logic memory, figure savings, Symbol-Digit-Modalities Test, incidental learning, Face Name Test); (2) Language (comp. of verbal fluency; groceries and animals, Boston naming test, FCSRT naming); (3) Executive functioning (comp. of Trail Making Test A + B, number cancelation, SDMT, Flanker Task); (4) Working memory (comp. of Digit Span Forward + Backward, FCSRT: interference task); (5) Visuospatial functioning (comp. of Clock copying + drawing, CERAD figure copying).	β (95% CI) (1) Memory: **0.045 (0.003, 0.087)** (2) Language: 0.039 (−0.002, 0.079) (3) Executive function: 0.014 (−0.029, 0.057) (4) Working memory: 0.031 (−0.014, 0.076) (5) Visuospatial functioning: 0.014 (−0.024, 0.052)	Age, sex, education, APOe4- status, total daily energy intake, BMI, smoking status, physical activity	Good
Escher (2022) [42]	UCSF Memory and Aging Center's Longitudinal Brain Aging Program (USA)	132	Older adults (mean age 71.7 ± 19y), free of dementia	15-MIND diet adherence (continuous) based on FFQ	Cognition measured by (1) Episodic memory (California Verbal Learning Test - long delay); (2) Executive function (Stroop interference, Digit Span Backwards, phonemic fluency, D-KEFS design fluency, modified trail making test); (3) Language (Boston naming test, category fluency)	β (95% CI) (1) Episodic memory: 0.03 (−0.01, 0.08) (2) Executive function: 0.15 (−0.03,0.33) (3) Language: 0.18 (−0.001, 0.04)	Age, sex, education, vascular burden score, PASE, MIND*PASE	Poor
Gauci (2022) [33]	Memory and Attention Supplement Trial cohort (Australia)	141	Middle-aged adults (40-65y), free of dementia	15-MIND diet adherence (continuous) based on multiple (2–4) 24 h recalls	Cognition measured by computer-based tests (1) Reaction and decision speed (comp. of simple reaction time, choice reaction time); (2) Visual processing (comp. of immediate recognition, delayed recognition, contextual memory task); (3) Stroop processing (comp. of difference incongruent and congruent stroop tasks); (4) Spatial working memory (comp. of 14 spatial working memory trials)	β (95% CI/SD/SE not shown) (1) Reaction and decision speed: −0.06, N·S. (2) Visual processing: −0.12, N·S. (3) Stroop processing: **0.19, p**<**0.035** (4) Spatial working memory: −0.13, N·S.	Age, sex, education, energy intake	Good
Zare (2023) [34]	No name (Iran)	60	Older adults (≥60y) with T2DM, free of dementia	14-MIND diet adherence (continuous) based on a MIND dietary scoring questionnaire	Cognition measured by (1) Stroop task 1 (time) (2) Stroop task 1 (errors) (3) Stroop task 2 (time) (4) Stroop task 2 (errors) (5) Trail making task (6) Forward digit span (7) Letter digit modality task (total) (8) Letter digit modality task (true responses)	r (p-value) (1) Stroop task 1 (time): −0.217 (n.s.) (2) Stroop task 1 (errors): −0.164 (n.s.) (3) Stroop task 2 (time): 0.025 (n.s.) (4) Stroop task 2 (errors): −0.092 (n.s.) (5) Trail making task: −0.165 (n.s.) (6) Forward digit span: 0.194 (n.s.) (7) Letter digit modality task (total): 0.247 (0.057) (8) Letter digit modality task (true responses): 0.245 (0.060)	None	Poor
Huang (2022) [45]	Chinese Longitudinal Healthy Longevity Study (China)	11245	Older adults (mean age 84 ± 11y) without stroke or dementia	12-MIND diet adherence (tertiles and continuous) based on simplified FFQ	Cognition measured by MMSE	β (95% CI) T1 vs T2: **0.60 (0.37, 0.82)** T1 vs T3: 1.01 (0.76, 1.26)	Sex, age, region, education, BMI, smoking, drinking, exercise, social engagement, hypertension, diabetes, depression, hearing impairment.	Fair
Vassilo-poulou (2022) [36]	No name (Greece)	167 (115 dementia; 52 cognitively healthy)	Older adults (mean age 72.6 ± 8.1 (dementia); 70.2 ± 4.6 (healthy))	9-MIND diet adherence (continuous) based on dietitian interview	Cognition measured by MMSE	β (r) (95% CI/SD/SE not shown): **0.24 (0.32), p**<**0.001**	Sex, age, BMI, DASS-21	Poor
Calil (2018) [43]	No name (Brazil)	96 (36 cognitively healthy, 30 MCI, 30 AD)	Older adults (≥60y) from neurology outpatient clinics	15-MIND diet adherence (tertiles) based on 98-item FFQ	Cognition measured by (1) MMSE; (2) Learning score of Brief Cognitive Screening Battery	β (95% CI) in cognitively healthy participants (1) MMSE T1 vs T2: **3.21 (0.95, 5.48), p = 0.007** T1 vs T3: 1.51 (−0.78, 3.79), p = 0.188 (2) Learning score Brief Cognitive Screening Battery T1 vs T2: 0.46 (−0.66, 1.60), p = 0.404 T1 vs T3: **1.39 (0.30, 2.49), p** = **0.014** *No association in MCI and AD patients (data not shown)*	(1) Age, education, partner, MedDiet score (2) Age, partner, MedDiet score	Poor
Yeung (2022) [44]	MrOs and MsOs study (Hong Kong/China)	3730	Older adults (≥65y)	9-MIND diet adherence (continuous) based on 280-item FFQ	Cognition (low/high performance based on median split of 4 items of MMSE; orientation to date, orientation to address, registration of three objects, and attention and calculation)	OR (95% CI) In men: 0.98 (0.88, 1.10), p = 0.743 In women: 1.00 (0.89, 1.14), p = 0.946	Age, BMI, education level, subjective social status, PASE score, daily energy intake, current smoker status, current alcohol use, number of chronic diseases.	Good

Abbreviations: AD: Alzheimer's disease, BMI: Body mass index, CERAD: Consortium to Establish a Registry for Alzheimer's disease, CI: Confidence Interval, comp.: composition score, CVD: cardiovascular disease, DASS-21: Depression Anxiety Stress Scale, D-KEFS: Delis-Kaplan Executive Function System, FCSRT: Free and Cued Selective Reminding Test, MCI: mild cognitive impairment, MedDiet: Mediterranean diet, MIND: Mediterranean-Dietary Approaches to Systolic Hypertension (DASH) diet Intervention for Neurodegenerative Delay, MMSE: Mini-Mental-State Examination, OR: odds ratio, PASE: Physical Activity Scale for the Elderly, SDMT: Symbol Digit Modalities Test, SE: standard error, sFFQ: simplified Food Frequency Questionnaire, TICS: Telephone Interview for Cognitive Status, TMT: Trail Making Test, T2D: Type 2 diabetes.

1 Study quality was assessed with the Newcastle Ottawa Scale.

**TABLE 2:** Description of included longitudinal studies describing the association between MIND diet and cognitive decline

Author (year)	Study (country)	Duration (years)	Sample size (n)	Population	Exposure	Outcome	Results	Covariates	Study quality^1^
Vu (2022) [20]	Chicago Health and Aging Project (USA)	Not shown	2449 (946 white, 1503 black)	Older adults (≥65y), free of dementia, either white or African (black) Americans	15-MIND diet adherence (tertiles, continuous) based on sFFQ	Change in global cognition (comp. of east Boston story immediate and delayed, symbol digit modalities test, MMSE)	β (95% CI) in white participants: T1 vs T2: 0.0001 (−0.01, 0.01), p = 0.99 T1 vs T3: −0.0008 (−0.01, 0.01), p = 0.89 Continuous: −0.004 (−0.003, 0.002), p = 0.78 β (95% CI) in black participants: T1 vs T2: 0.0003 (−0.01, 0.01), p = 0.95 T1 vs T3: −0.003 (−0.01, 0.01), p = 0.51 Continuous: −0.00002 (−0.003, 0.003), p = 0.99	Age, sex, study centre, education, income, global cognition score, late life cognitive activity, history of diabetes, hypertension, stroke, heart disease, smoking, calorie intake, BMI, depressive symptoms, physical activity	Good
Vu (2022) [20]	Rush Memory and Aging Project (USA)	Not shown	725	Older adults (mean age 82y), free of dementia	15-MIND diet adherence (tertiles, continuous) based on sFFQ	Change in global cognition (comp of word list memory, recall and recognition, east Boston story immediate and delayed, logical memory IIa immediate and delayed, Boston naming test, verbal fluency, reading test, digit span forward and backward, digit ordering, symbol digit modalities test, number comparison, stroop word reading and colour naming, judgement of line orientation, standard progressive matrices)	β (95% CI) T1 vs T2: 0.006 (−0.01, 0.02), p = 0.50 T1 vs T3: **0.03 (0.01, 0.05), p** = **0.001** Continuous: **0.006 (0.003, 0.01), p** = **0.002**	Age, sex, study centre, education, income, global cognition score, late life cognitive activity, history of diabetes, hypertension, stroke, heart disease, smoking, calorie intake, BMI, depressive symptoms, physical activity	Good
Cherian (2019) [22]	Rush Memory and Aging Project (USA)	5.9 (mean follow-up)	106	Older adults (mean age 82.8y) with a clinical history of stroke and no dementia	15-MIND diet adherence (tertiles) based on 144-item sFFQ	Change in cognition measured by (1) Global cognition (comp. of all domains) (2) Episodic memory (comp. of word list, word list recall/recognition, East Boston immediate/delayed recall, logic memory immediate/delayed) (3) Semantic memory (comp. of Boston naming test, category fluency, reading test) (4) Working memory (comp. of digits forward, digits backwards, digit ordering) (5) Visuospatial memory/perceptual orientation (comp. of line orientation, progressive matrices) (6) Perceptual speed (comp. of symbol digits modality, number comparison, stroop colour naming, stroop word reading)	β (95% CI) (1) Global cognition: T1 vs T2: 0.058 (−0.011, 0.128) T1 vs T3: **0.083 (0.007, 0.158)** (2) Episodic memory: T1 vs T2: 0.025 (−0.048, 0.098) T1 vs T3: 0.041 (−0.038, 0.121) (3) Semantic memory T1 vs T2: 0.030 (−0.033, 0.093) T1 vs T3: **0.070 (0.001, 0.138)** (4) Working memory T1 vs T2: 0.023 (−0.041, 0.087) T1 vs T3: 0.033 (−0.037, 0.102) (5) Visuospatial memory T1 vs T2: 0.062 (−0.001, 0.126) T1 vs T3: 0.061 (−0.008, 0.130) (6) Perceptual speed T1 vs T2: 0.047 (−0.019, 0.113) T1 vs T3: **0.071 (0.000, 0.142)**	Age, sex, education, APOE4, caloric intake, smoking, participation in cognitive and physical activity	Good
Morris (2015) [4]	Rush Memory and Aging Project (USA)	4.7 (mean follow-up)	835 - 860 (depending on outcome measure)	Older adults (mean age 81.4 ± 7.2y), free of dementia	15-MIND diet adherence (continuous) based on 144-item sFFQ	Change in cognition measured by (1) Global cognition (comp. of all domains) (2) Episodic memory (comp. of word list memory, recall and recognition, East Boston story immediate and delayed recall, story A from logical memory of Wechsler memory scale-revised) (3) Working memory (comp. of digit span forward and backward, digit ordering) (4) Semantic memory (comp. of Boston naming test, verbal fluency, national adult reading test) (5) Visuospatial ability (comp. of judgement of line orientation, standard progressive matrices) (6) Perceptual speed (comp. of symbol digit modalities test, number comparison, stroop test)	β ± SE (1) Global cognition: **0.0106 ± 0.0023, p**<**0.0001** (2) Episodic memory: **0.0090 ± 0.0028, p** = **0.001** (3) Working memory: **0.0060 ± 0.0024, p** = **0.01** (4) Semantic memory: **0.0113 ± 0.0027, p**<**0.0001** (5) Visuospatial ability: **0.0077 ± 0.0025, p** = **0.002** (6) Perceptual speed: **0.0097 ± 0.0023, p**<**0.0001**	Age at first cognitive assessment, sex, education, participation in cognitive activities, APOE4, smoking history, physical activity hours per week, total energy intake, time, history of stroke, myocardial infarction, diabetes, hypertension, interaction terms between time and each model covariate, MIND diet score	Good
Dhana (2021) [24]	Memory and Aging Project (USA)	Not shown	569	Older adults (mean age at death 90.8 ± 6.1y), some were diagnosed with AD	15-MIND diet adherence (continuous, per 1SD = 1.42 point) based on 144-item sFFQ	Change in global cognition proximate to death (comp. of East Boston Story immediate/delayed recall, Story A from Logical Memory, Word List Memory, Word List Recall/recognition, Boston Naming Test, Verbal Fluency, Word reading test, Digit Span Forward/Backward, Digit Ordering)	β ± SE **0.119** ± **0.040, p** = **0.003**	Age at death, sex, education, APOE4, late-life cognitive activities, total energy intake.	Poor
Van Lent (2021) [28]	Framingham Heart Offspring Study (USA)	6.6 ± 1.1 (mean follow-up)	2092	Older adults (mean age 61 ± 9y), free of dementia	15-MIND diet adherence (continuous) based on 126-item sFFQ	Change in cognition measured by (1) Visual reproductions delayed recall (2) Logical memory delayed recall (3) Trail making test (TMT) A (4) Trail making test B/A (5) Hooper visual organization test (6) Similarities (7) Global cognition (comp. of tests above)	β ± SE (1) Visual reproductions delayed recall: −0.01 ± 0.02, p = 0.58 (2) Logical memory delayed recall: −0.02 ± 0.02, p = 0.32 (3) TMT A: −0.004 ± 0.02, p = 0.79 (4) TMT B/A: −0.02 ± 0.02, p = 0.28 (5) Hooper visual organization test: −0.02 ± 0.02, p = 0.31 (6) Similarities: 0.03 ± 0.02, p = 0.05 (7) Global cognition: −0.002 ± 0.02, p = 0.87	Age, age squared, sex, ApoE4 status, total energy intake, education, BMI, physical activity, smoking, diabetes, CVD, depressive symptoms, anti-hypertensive medication, systolic blood pressure, total cholesterol to HDL ratio, time interval between FFQ and outcome measure.	Good
Berendsen (2018) [39]	Nurses' Health Study (USA)	6	16058	Older women (≥70y), free of stroke and dementia	15-MIND diet adherence (quantiles) based on 116-item sFFQ	Change in cognition measured by (1) TICS (2) Verbal (episodic) memory (comp. of immediate and delayed recalls of the East Boston Memory Test and delayed recall of the TICS) (3) Global cognition (comp. of aforementioned tests and category fluency digit span backward)	Mean difference (95% CI) (1) TICS Q1 vs Q2: 0.14 (−0.018, 0.045) Q1 vs Q3: 0.003 (−0.030, 0.035) Q1 vs Q4: −0.011 (−0.043, 0.020) Q1 vs Q5: 0.004 (−0.028, 0.036) (2) Verbal memory Q1 vs Q2: 0.000 (−0.009, 0.009) Q1 vs Q3: −0.007 (−0.017, 0.002) Q1 vs Q4: −0.003 (−0.013, 0.006) Q1 vs Q5: 0.002 (−0.008, 0.011) (3) Global cognitive score Q1 vs Q2: 0.001 (−0.0007, 0.009) Q1 vs Q3: −0.004 (−0.011, 0.004) Q1 vs Q4: −0.002 (−0.010, 0.006) Q1 vs Q5: 0.001 (−0.007, 0.009)	Age, education, physical activity, calorie intake, alcohol intake, smoking status, multivitamin use, BMI, depression, history of high blood pressure, hypercholesterolemia, myocardial infarction, diabetes mellitus.	Fair
Boumenna (2022) [40]	The Boston Puerto Rican Health Study (USA)	8	573	Middle to older-aged adults (45-75y)	15-MIND diet adherence (quantiles and continuous) based on FFQ	Change in global cognition (comp. of MMSE, 16 word list learning, digit span forward and backward, stroop test, clock drawing and figure copying, verbal fluency)	β (95% CI) Q1 vs Q2: 0.005 (−0.053, 0.064) Q1 vs Q3: 0.006 (−0.043, 0.055) Q1 vs Q4: 0.047 (−0.006, 0.099) Q1 vs Q5: **0.093 (0.035, 0.152)** Continuous: **0.0213 (0.008, 0.034), p** = **0.0013**	Age, sex, BMI, physical activity score, diabetes, hypertension, education level, smoking, alcohol use, ApoE4 carrier, energy intake, job complexity score, poverty index	Good
Nishi (2021) [46]	PREvención con DIeta MEDiterránea-Plus trial (Spain)	2	5714	Older adults (55-75y) with overweight or obesity and metabolic syndrome	15-MIND diet adherence (tertiles) based on 143-item sFFQ	Change in cognition measured by (1) Global cognition (comp. of test below) (2) MMSE (3) Clock drawing test (4) Verbal fluency semantical (5) Verbal fluency phonological (6) TMT A (7) TMT B (8) Digit span forward (9) Digit span backward	β (95% CI) (1) Global cognition T1 vs T2: −0.020 (−0.057, 0.016) T1 vs T3: 0.023 (−0.017, 0.063) (2) MMSE T1 vs T2: 0.044 (−0.007, 0.095) T1 vs T3: 0.039 (−0.014, 0.092) (3) Clock drawing test T1 vs T2: 0.002 (−0.056, 0.060) T1 vs T3: 0.030 (−0.030, 0.090) (4) Verbal fluency semantical T1 vs T2: −0.003 (−0.051, 0.045) T1 vs T3: −0.036 (−0.086, 0.014) (5) Verbal fluency phonological T1 vs T2: −0.030 (−0.077, 0.018) T1 vs T3: 0.015 (−0.035, 0.064) (6) TMT A T1 vs T2: 0.023 (−0.031, 0.076) T1 vs T3: −0.017 (−0.077, 0.044) (7) TMT B T1 vs T2: 0.045 (−0.003, 0.094) T1 vs T3: 0.022 (−0.031, 0.075) (8) Digit span forward T1 vs T2: −0.043 (−0.095, 0.009) T1 vs T3: −0.007 (−0.065, 0.051) (9) Digit span backward T1 vs T2: 0.006 (−0.045, 0.057) T1 vs T3: 0.055 (−0.001, 0.112	Age, sex, intervention group, centre size, corrected for clusters, respective cognitive test score at baseline, baseline education level, civil status, smoking habits, BMI, hypertension, hypercholesterolemia, diabetes, and depressive symptomology, baseline physical activity, and total energy intake.	Good
Lotan (2022) [47]	Israel Diabetes and Cognitive Decline study (Israel)	4.1 ± 2.1 (mean follow-up)	960	Older adults (≥65y) with T2DM, free of dementia	15-item MIND diet adherence (continuous) based on FFQ	Change in cognition measured by (1) Global cognition (comp. of all domains) (2) Episodic memory (comp. of word list immediate, delayed and recognition) (3) Attention/working memory (comp. of shape cancellation, digit span forward and backward) (4) Language/semantic categorization (comp. of similarities, animal fluency and 15-item boston naming test) (5) Executive function (comp. of TMT A and B, praxis, and digit symbol substitution test)	β±SE (1) Global cognition: 0.00604 ± 0.00354, p = 0.087 (2) Episodic memory: 0.00219 ± 0.00584, p = 0.707 (3) Attention: 0.00030 ± 0.0054, p = 0.954 (4) Language: 0.00559 ± 0.00374, p = 0.135 (5) Executive function: **0.00978** ± **0.00446, p** = **0.028**	Age, sex, education, daily calories, duration of T2D at baseline, baseline cholesterol, creatinine, HbA1c, triglycerides, systolic blood pressure, diastolic blood pressure, BMI, diabetic medication, physical activity	Good
Huang (2023) [12]	China Health and Nutrition Survey (China)	3 (median follow-up)	4066	Older adults (≥55y), free of dementia	12-MIND diet adherence (tertiles and continuous) based on 3 24 h dietary recalls	Change in cognition measured by (1) Global cognition (comp. of items of TICS-m) (2) Verbal memory (comp. of immediate and delayed recall)	β (95% CI) (1) Global cognition T1 vs T2: **0.016 (0.004, 0.029)** T1 vs T3: 0.010 (−0.003, 0.023) Continuous (per 3 points): 0.006 (−0.009, 0.020) (2) Verbal memory T1 vs T2: 0.012 (−0.001, 0.025) T1 vs T3: 0.007 (−0.006, 0.021) Continuous (per 3 points): 0.004 (−0.011, 0.019)	Age, age square, sex, education, residence, region, income, smoking status, drinking status, BMI, total energy, physical activities, hypertension, diabetes, myocardial infarction	Fair
Dong (2023) [35]	Wisconsin Registry for Alzheimer's Prevention (USA)	Not shown	1078	Older adults (mean age 63.5 ± 6.7y), free of dementia	15-MIND diet adherence (continuous) based on 15-item self-reported diet questionnaire	Change in cognition measured by (1) Preclinical Alzheimer cognitive composite (PACC) (2) Immediate learning (Rey auditory verbal learning test total trials 1–5, Wechsler memory scale–revised logical memory subtest immediate recall, and brief visuospatial memory test immediate recall)	β (p-value) (1) PACC: 0.0087 (0.388). (2) immediate learning: −0.0038 (0.770). *Data on delayed recall and executive function were also available but no effect sizes were given*	None	Poor
Munoz-Garcia (2020) [48]	Seguimiento Universidad de Navarra Project (Spain)	6	806	Older adults (>55y), free of dementia	15-MIND diet adherence (tertiles and continuous) based on 136-item sFFQ	Change in cognition measured by STICS-m	β (95% CI) T1 vs T2: 0.17 (−0.28, 0.62) T1 vs T3: 0.47 (−0.07,1.02) Continuous (per 1SD/1.5 points): **0.27 (0.05. 0.48)**	Age at time baseline STICS-m, sex, follow-up time until baselines STICS-m, years of university education, APOE4, smoking status, package-years, total energy intake, physical activity, BMI, alcohol intake, hypertension, high cholesterol, low HDL, and prevalent disease at recruitment (depression, cardiovascular disease, and diabetes).	Fair
Shakersain (2018) [49]	The Swedish National Study on Aging and Care in Kungsholmen (Sweden)	6	2223	older adults (≥60y), free of dementia	14-MIND diet adherence (continuous and tertiles) adapted to range 0–66, based on 98-item sFFQ	(1) Change in cognition measured by MMSE (2) Risk of cognitive decline, defined as MMSE score of ≤24 after 6y	(1) β (95% CI) for change in MMSE T1 vs T2: **0.075 (0.012, 0.138), p** = **0.019** T1 vs T3: **0.126 (0.064, 0.188), p**<**0.001** Continuous: **0.006 (0.003, 0.009), p**<**0.001** (2) HR (95% CI) for risk of cognitive decline T1 vs T2: 0.781 (0.494, 1.235) p = 0.289 T1 vs T3: **0.468 (0.261, 0.840) p** = **0.011** Continuous: **0.965 (0.941, 0.989) p** = **0.005**	Total calorie intake, age, sex, education, civil status, physical activity, smoking, body mass index, vitamin/mineral supplement intake, vascular disorders, diabetes, cancer, ApoE4, dietary components other than main exposures	Poor

Abbreviations: AD: Alzheimer's disease, BMI: Body mass index, CI: Confidence Interval, comp.: composition score, CVD: cardiovascular disease, HR: Hazard Ratio, MIND: Mediterranean-Dietary Approaches to Systolic Hypertension (DASH) diet Intervention for Neurodegenerative Delay, MMSE: Mini-Mental-State Examination, PACC: Preclinical Alzheimer cognitive composite, SE: standard error, sFFQ: simplified Food Frequency Questionnaire, TICS: Telephone Interview for Cognitive Status, TMT: Trail Making Test, T2D: Type 2 diabetes.

1 Study quality was assessed with the Newcastle Ottawa Scale.

**TABLE 3:** Description of included studies describing the association between MIND diet adherence and dementia

Author (year)	Study (country)	Duration (years)	Sample size (n)	Population	Exposure	Outcome	Results	Covariates	Study quality^1^
Vassilo-poulou (2022) [36]	No name (Greece)	N.A.; case-control	167 (115 dementia; 52 cognitively healthy controls)	Older adults; either dementia (mean age 72.6 ± 8.1) or cognitively healthy (mean age 70.2 ± 4.6)	9-MIND diet adherence (continuous) based on dietitian interview	Odds of dementia	OR (95% CI) **0.43 (0.29, 0.63)**	Sex, age, BMI, DASS-21, MMSE	Poor
Filippini (2020) [52]	No name (Italy)	N.A.; case-control	108 (n = 54 cases)	Early onset dementia patients (cases) and caregivers (controls) (mean age 65y)	15-MIND diet adherence (continuous, tertiles) based on 188-item sFFQ	Odds of (1) Early onset dementia (EOD) (2) Early onset AD (EO-AD) (3) Early onset frontotemporal dementia spectrum (EO-FTD)	OR (95% CI) (1) EOD: T1 vs T2: **0.32 (0.12, 0.83)** T1 vs T3: **0.31 (0.11, 0.90)** Continuous: **0.66 (0.47, 0.91)** (2) EO-AD: T1 vs T2: 0.39 (0.13, 1.15) T1 vs T3: 0.32 (0.09, 1.13) Continuous: **0.67 (0.46, 0.98)** (3) EO-FTD: T1 vs T2: 0.31 (0.07, 1.28) T1 vs T3: 0.45 (0.10, 2.00) Continuous: 0.66 (0.41, 1.08)	Sex, age, educational attainment, total energy intake	Poor
Thomas (2022) [37]	The Three-City Bordeaux study (France)	9.7	1412	Older adults (mean age 75.8 ± 4.8), free of dementia	15-MIND diet adherence (tertiles and continuous) based on 148-item FFQ and one 24 h recall	Incident (1) All-cause dementia (2) AD	HR (95% CI) (1) All-cause dementia: T1 vs T2: 0.93 (0.73, 1.17) T1 vs T3: **0.73 (0.55, 0.97)** Continuous: **0.90 (0.83, 0.96), p** = **0.003** (2) AD: T1 vs T2: 0.96 (0.72, 1.27) T1 vs T3: 0.70 (0.49, 1.00) Continuous: **0.89 (0.81, 0.97), p** = **0.008**	Sex, APOE4 status, educational level, total energy intake, BMI, tobacco consumption, practice of regular physical activity, diabetes, history of cerebral and cardiovascular disease, hypertension, hypercholesterolemia, depressive symptoms (age as time scale)	Good
Morris (2015) [5]	Rush Memory and Aging Project (USA)	4.5	789	Older adults (58-98y), free of AD	15-MIND diet adherence (tertiles) based on 144-item sFFQ	Incident AD	HR (95% CI) T1 vs T2: **0.64 (0.42, 0.97)** T1 vs T3: **0.48 (0.29, 0.79)** p for trend = **0.003**	Age, sex, education, APOE4, participation in cognitively stimulating activities, physical activity, total energy intake, cardiovascular conditions (hypertension, myocardial infarction, diabetes, stroke)	Good
Vu (2022) [20]	Chicago Health and Aging Project (USA)	not shown	2449 (946 white, 1503 black)	Older adults (≥65y), either white or African (black) Americans, free of dementia	15-MIND diet adherence (tertiles, continuous) based on sFFQ	Incident all-cause dementia	OR (95% CI) in white participants T1 vs T2: 0.87 (0.30, 2.54), p = 0.80 T1 vs T3: 1.23 (0.47, 3.18), p = 0.68 Continuous: 1.00 (0.81, 1.25), p = 0.97 OR (95% CI) in black participants T1 vs T2: 0.86 (0.36, 2.05), p = 0.74 T1 vs T3: 1.48 (0.51, 4.27), p = 0.47 Continuous: 1.08 (0.79, 1.48), p = 0.61	Age, sex, study centre, education, income, global cognition score, late life cognitive activity, history of diabetes, hypertension, stroke, heart disease, smoking, calorie intake, BMI, depressive symptoms, physical activity	Good
Vu (2022) [20]	Rush Memory and Aging Project (USA)	not shown	725	Older adults (mean age 82y), free of dementia	15-MIND diet adherence (tertiles and continuous) based on sFFQ	Incident all-cause dementia	HR (95% CI) T1 vs T2: 0.85 (0.62, 1.16), p = 0.31 T1 vs T3: **0.63 (0.42, 0.92), p** = **0.02** Continuous: 0.91 (0.83, 1.00), p = 0.06	Age, sex, study centre, education, income, global cognition score, late life cognitive activity, history of diabetes, hypertension, stroke, heart disease, smoking, calorie intake, BMI, depressive symptoms, physical activity	Good
Vu (2022) [20]	Women's Health Initiative Memory Study (USA)	not shown	5308	Older female (≥65y), free of dementia	15-MIND diet adherence (tertiles and continuous) based on sFFQ	Incident all-cause dementia	HR (95% CI) T1 vs T2: **0.87 (0.79, 0.97), p** = **0.008** T1 vs T3: **0.80 (0.72, 0.89), p**<**0.0001** Continuous: **0.95 (0.92, 0.97), p**<**0.0001**	Age, study centre, randomization status, education, income, global cognition score, history of diabetes, hypertension, stroke, heart disease, smoking, calorie intake, BMI, depressive symptoms, physical activity	Good
de Crom (2022) [50]	Rotterdam Study (Netherlands)	15.6 (baseline 1) 5.9 (baseline 2)	5375 (baseline 1) 2861 (baseline 2)	Older adults (≥55y), free of dementia	15-MIND diet adherence (continuous) based on 170-item (baseline 1) or 389-item FFQ (baseline2)	Incident all-cause dementia	HR (95% CI) baseline 1: 0.99 (0.94, 1.05) baseline 2: **0.79 (0.70, 0.91)**	Sex, age, age^2^, educational attainment, smoking status, physical activity, daily energy intake, BMI, diabetes, hypercholesterolemia, hypertension.	Good
Hosking (2019) [51]	The 60's cohort of the Personality and Total Health Through Life (Australia)	12	961	Older adults (60-64y), free of dementia	13-MIND diet adherence (continuous) based on 183-item sFFQ	Incident dementia	OR (95% CI) **0.72 (0.54, 0.95)**	Age, sex, energy intake	Fair
Cornelis (2023) [29]	UK Biobank (UK)	10.5 ± 1.8 (mean follow-up)	77398	Older adults ≥55y, free of dementia	15-MIND diet adherence (tertiles and continuous) based on 1 to 4 Oxford webQs (web-based 24 h dietary assessment tool)	Incident (1) All-cause dementia (2) Alzheimer's dementia	HR (95% CI) (1) All cause dementia T1 vs T2: 1.06 (0.90, 1.24) T1 vs T3: 0.90 (0.74, 1.09) Continuous 0.99 (0.95, 1.03) (2) Alzheimer's dementia T1 vs T2: 1.00 (0.78, 1.30) T1 vs T3: 0.96 (0.72, 1.28) Continuous: 1.01 (0.95, 1.07)	Age, sex, self-reported race/ethnicity, education, Townsend deprivation index, income, employment status, global cognition score, family history of dementia; history of hypertension, diabetes, heart disease, stroke and depression; self-reported health, smoking, physical activity, BMI, fast meal consumption and energy intake	Fair
Zhang (2023) [30]	UK Biobank (UK)	9.4	114684	Middle-aged to older adults (40-69y), free of dementia	14-MIND diet adherence (tertiles and continuous) based on 2–4 Oxford web-based 24 h dietary assessment tool, scored according to quintiles of intake	Incident dementia	HR (95% CI) T1 vs T2: 0.91 (0.73, 1.14) T1 vs T3: 0.89 (0.71, 1.12)	Age, sex, educational level, Townsend deprivation index, BMI, smoking status, alcohol consumption, regular physical activity, sleep duration, time on watching TV, family history of AD, APOE genotypes, cancer, CVD, diabetes	Good
Chen (2023) [11]	Whitehall II study (UK)	12.9	8358	Older adults (≥45y), free of dementia	14-MIND diet adherence (tertiles and continuous), rescaled to 15-points, based on FFQ	Incident dementia	HR (95% CI) T1 vs T2: 1.03 (0.73, 1.45) T1 vs T3: 0.96 (0.66, 1.38) Continuous (per 3 points): 0.97 (0.72, 1.30)	Age, sex, education level, occupational class, vigorous physical activity, smoking status, energy intake, BMI, depressive symptoms, hypertension, hypercholesterolemia, diabetes, stroke, cardiovascular diseases	Good
Chen (2023) [11]	Health and Retirement Study (USA)	5.0	6758	Older adults (≥45y), free of dementia	15-MIND diet adherence (tertiles and continuous) based on FFQ	Incident dementia	HR (95% CI) T1 vs T2: 0.95 (0.73, 1.25) T1 vs T3: 0.83 (0.63, 1.09) Continuous (per 3 points): **0.82 (0.68**–**0.99)**	Age, sex, education level, household income, vigorous physical activity, smoking status, energy intake, BMI, depressive symptoms, hypertension, diabetes, stroke, cardiovascular diseases	Good
Chen (2023) [11]	Framingham Heart Study, Offspring cohort (USA)	10.7	3020	Older adults (≥45y), free of dementia	15-MIND diet adherence (tertiles and continuous) based on FFQ	Incident dementia	HR (95% CI) T1 vs T2: 0.96 (0.70, 1.33) T1 vs T3: **0.69 (0.48, 0.99)** Continuous (per 3 points): 0.76 (0.57, 1.00)	Age, sex, education level, household income, vigorous physical activity, smoking status, energy intake, BMI, depressive symptoms, hypertension, hypercholesterolemia, diabetes, stroke, cardiovascular diseases	Good

Abbreviations: AD: Alzheimer's disease, BMI: Body mass index, CI: Confidence Interval, CVD: cardiovascular disease, DASS-21: Depression Anxiety Stress Scale, EO-AD: Early onset Alzheimer's disease, EOD: Early onset dementia, EO-FTP: Early onset frontotemporal dementia spectrum HR: Hazard Ratio, MIND: Mediterranean-Dietary Approaches to Systolic Hypertension (DASH) diet Intervention for Neurodegenerative Delay, MMSE: Mini-Mental-State Examination, OR: Odds Ratio, sFFQ: simplified Food Frequency Questionnaire.

1 Study quality was assessed with the Newcastle Ottawa Scale.

**TABLE 4:** Description of included studies describing the association between MIND diet adherence and cognitive impairment, subjective memory complaints, and cognitive resilience

Author (year)	Study (country)	Design	Duration (years)	Sample size (n)	Population	Exposure	Outcome	Results	Covariates	Study quality^1^
Lawrie (2022) [53]	Oxford Parkinson's Disease Discovery Cohort (UK)	Cross-sectional	N.A.	131	Older adults (67±9y) with Parkinson's disease, free of dementia	15-MIND diet adherence (continuous) based on FFQ	Odds of mild cognitive impairment (MoCA, adjusted for education)	β (95% CI/SD/SE not shown) −0.23, p = 0.070	Age, sex, kcal, disease duration, physical activity level, education, smoking status	Poor
Huang (2022) [45]	Chinese Longitudinal Healthy Longevity Study (China)	Cross-sectional	N.A.	11245	Older adults (84 ± 11y) without stroke or dementia	Chinese-adapted 12-MIND diet adherence (tertiles and continuous) based on sFFQ	Odds of cognitive impairment (MMSE, adjusted for education)	OR (95% CI) T1 vs T2: **0.81 (0.71, 0.92)** T1 vs T3: **0.60 (0.51, 0.72)** Continuous: **0.86 (0.82, 0.89)**	Sex, age, region, education, BMI, smoking, drinking, exercise, social engagement, hypertension, diabetes, depression, hearing impairment.	Fair
Hosking (2019) [51]	The 60's cohort of the Personality and Total Health Through Life (Australia)	Long-itudinal	12	961	Older adults (60-64y), free of dementia	13-MIND diet adherence (tertiles and continuous) based on 183-item sFFQ	Incident MCI (Winbald criteria)	OR (95% CI) T1 vs T2: 0.94 (0.57, 1.56) T1 vs T3: **0.47 (0.24, 0.91)** p for trend: **0.026**	Energy intake, age, sex, APOE4 status, education, mental activity, physical activity, smoking status, depression, diabetes, BMI, hypertension, heart disease, stroke	Good
Adjibade (2019) [32]	NutriNet-Santé cohort (France)	Long-itudinal	6	6011	Older adults (≥60y), free of dementia	15-MIND diet adherence (tertiles and continuous), based on 3 non-consecutive 24 h dietary records	Subjective memory complaints (SMC) (cognitive difficulty scale (CDS), cut-off score of 43)	HR (95% CI) total population T1 vs T2: 0.97 (0.84, 1.12) T1 vs T3: 0.94 (0.79, 1.11) Continuous: 0.98 (0.93, 1.02), p = 0.32 HR (95% CI) 60-69y T1 vs T2: 1.00 (0.85, 1.18) T1 vs T3: 0.97 (0.80, 1.17) Continuous: 1.00 (0.95, 1.05), p = 0.96 HR (95% CI) ≥70y T1 vs T2: 0.84 (0.60, 1.17) T1 vs T3: 0.81 (0.55, 1.20) Continuous: **0.87 (0.78, 0.98), p** = **0.02**	Age, sex, material status, educational level, occupational category, household income per consumption unit, energy intake without alcohol, number of recording days, inclusion moth, smoking status, physical activity, BMI, comorbid conditions during follow-up, depressive symptoms at the end of the follow-up, baseline CDS score.	Good
Wagner (2023) [25]	Memory and Ageing Project (USA)	Long-itudinal	9 ± 4	578	Older adults (mean age diet assessment 84.1 ± 5.8; death 91.4 ± 6.1), free of dementia	15-MIND diet adherence (tertiles and continuous) based on 144-item sFFQ	(1) Cognitive resilience mean level, based on change in global cognition (comp. of 17 tests) adjusted for neuropathologies. (2) Cognitive resilience slope, based on the slope of global cognitive decline given a specific profile of neuropathologies	Mean difference (95% CI) (1) Cognitive resilience mean level T1 vs T2: 0.23 **(0.04, 0.41) p** = **0.02** T1 vs T3: **0.34 (0.14, 0.55) p** = **0.001** Continuous: **0.07 (0.02, 0.12) p** = **0.01** (2) Cognitive resilience slope T1 vs T2: **0.20 (0.01, 0.39) p** = **0.04** T1 vs T3: **0.27 (0.05, 0.48) p** = **0.01** Continuous: 0.05 (−0.003, 0.10) p = 0.06	Sex, education, age at first dietary assessment, total energy intake, smoking status, number of depressive symptoms, number of medical conditions, physical activity, frequency of participation in cognitively stimulating activities	Good

Abbreviations: BMI: Body mass index, CDC: cognitive difficulty score, CI: Confidence Interval, comp.: composition score, HR: Hazard Ratio, MIND: Mediterranean-Dietary Approaches to Systolic Hypertension (DASH) diet Intervention for Neurodegenerative Delay, MMSE: Mini-Mental-State Examination, MoCA: Montreal Cognitive Assessment, OR: Odds Ratio, SD: standard deviation, SE: standard error, sFFQ: simplified Food Frequency Questionnaire, SMC: Subjective memory complaints.

1 Study quality was assessed with the Newcastle Ottawa Scale.

**TABLE 5:** Description of included studies describing the association between MIND diet adherence and Parkinson's disease

Author (year)	Study (country)	Design	Duration (years)	Sample size (n)	Population	Exposure	Outcome	Results	Covariates	Study quality^1^
Metcalfe-Roach (2021) [54]	no name (Canada)	Cross-sectional	N.A.	n = 121	Older adults (mean age 65y) with diagnosis of PD	15-MIND diet adherence (continuous) based on FFQ, MIND score adjusted to 0–10 scale	Age of PD onset	Beta (95% CI/SE/SD not shown) **2.2, p** = **0.002**	Disease duration, kcal, sex, smoking, years of education, exercise	Poor
Agarwal (2018) [21]	Rush Memory and Aging Project (USA)	Long-itudinal	4.6y (mean follow-up)	n = 706	Older adults (59-97y), free of PD and dementia	15-MIND diet adherence (tertiles, continuous) based on 114-item FFQ	(1) Incident PD (2) Change in PD progression	(1) HR (95% CI) for incident PD T1 vs T2 **0.70 (CI not shown) p** = **0.008** T1 vs T3 **0.58 (CI not shown) p** = **0.0003** Continuous **0.89 (0.83**–**0.96) p**<**0.05** (2) β (SE) for change in PD progression Continuous **−0.008 (0.0037), p = 0.04**	Age, sex, smoking, total energy intake, BMI, depressive symptoms	Poor

Abbreviations: BMI: Body Mass index, CI: Confidence Interval, FFQ: Food Frequency Questionnaire, HR: Hazard ratio, kcal: kilocalories, MIND: Mediterranean-Dietary Approaches to Systolic Hypertension (DASH) diet Intervention for Neurodegenerative Delay, PD: Parkinson's disease, SD: Standard deviation, SE: Standard error. ^1^ Study quality was assessed with the Newcastle Ottawa Scale.

**TABLE 6:** Description of included studies describing the association between MIND diet adherence and brain volume and pathology outcomes

Author (year)	Study (country)	Design	Duration (years)	Sample size (n)	Population	Exposure	Outcome	Results	Covariates	Study quality^1^
van Lent (2021) [28]	Framingham Heart Study, Offspring cohort (USA)	Cross-sectional	N.A.	1904	Older adults (61±9y), free of dementia	15-MIND diet adherence (continuous) based on 126-item sFFQ	Brain volume (% of intracranial volume) measured by (1) Total brain (2) Lateral ventricular (3) Hippocampal (4) White matter hyperintensity (5) Odds of silent brain infarcts	β (SE) (1) Total brain: **0.02 (0.01), p** = **0.02** (2) Lateral ventricular: −0.007 (0.01), p = 0.59 (3) Hippocampal: 0.02 (0.01), p = 0.20 (4) White matter hyperintensity: −0.02 (0.01), p = 0.15 OR (95% CI) (5) Silent brain infarcts: 0.99 (0.91, 1.09), p = 0.89	Age, age^2^, sex, ApoE4 status, total energy intake, education, BMI, physical activity, smoking, diabetes, CVD, depressive symptoms, anti-hypertensive medication, systolic blood pressure, total cholesterol to HDL ratio, time interval between FFQ and outcome measure.	Good
Dhana (2021) [24]	Rush Memory and Aging Project (USA)	Longitudinal	Not shown	569	Older adults (≥65y; mean age at death 90.8 ± 6.1y), some were diagnosed with AD	15-MIND diet adherence (continuous, per 1SD = 1.42 point) based on 144-item sFFQ	Brain pathology measured by (1) Global AD pathology (comp. of neurotic, diffuse plaques, and neurofibrillary tangles) (2) β-amyloid (3) Tangles (4) Macroinfarcts (5) Microinfarcts (6) Arteriolosclerosis (7) Cerebral atherosclerosis	β (SE) (1) AD pathology: −0.013 (0.024), p = 0.578 (2) β-amyloid: -0.03 (0.049), p = 0.395 (3) Tangles: 0.058 (0.332), p = 0.862 (4) Macroinfarcts: 0.038 (0.091), p = 0.680 (5) Microinfarcts: 0.132 (0.095), p = 0.163 (6) Arteriolosclerosis: 0.087 (0.098), p = 0.378 (7) Cerebral atherosclerosis: 0.033 (0.104), p = 0.754	Age at death, sex, education, APOE4, late-life cognitive activities, total energy intake.	Good
Agarwal (2023) [23]	Memory and Ageing Project (USA)	Longitudinal	6.8 ± 3.9y (mean follow-up)	581	Older adults (mean age diet assessment 84.2 ± 5.8; age death 91.3 ± 6.1)	15-MIND diet adherence (tertiles and continuous) based on 144-item sFFQ	Brain pathology measured by (1) Global AD pathology (2) Beta-amyloid load (3) Phosphorylated tau-tangle	β (SE) (1) Global AD pathology T1 vs T2: −0.027 (0.037) p = 0.461 T1 vs T3: −0.077 (0.038) p = 0.044 Continuous: −0.024 (0.011) p = 0.025 (2) Beta-amyloid load T1 vs T2: −0.099 (0.118) p = 0.402 T1 vs T3: −0.246 (0.123) p = 0.047 Continuous: −0.062 (0.034) p = 0.071 (3) Phosphorylated tau-tangle T1 vs T2: −0.139 (0.130) p = 0.285 T1 vs T3: −0.108 (0.134) p = 0.422 Continuous: −0.024 (0.037) p = 0.528	Age at death, sex, education, ApoE4 status, total calories, time between last dietary assessment and death	Good
Dong (2023)([35]	Wisconsin Registry for Alzheimer's Prevention (USA)	Longitudinal	Unknown	924	Older adults (mean age 63.5 ± 6.7y)	15-MIND diet adherence (continuous) based on 15-item diet questionnaire	Cerebrospinal fluid biomarkers (1) P-tau (2) T-tau	β (p-value) (1) P-tau: -0.1842 (0.37) (2) T-tau: -2.244 (0.31)	None	Poor
Escher (2022) [42]	UCSF Memory and Aging Center's Longitudinal Brain Aging Program (USA)	Cross-sectional	N.A.	77	Older adults (≥50y)	15-MIND diet adherence (continuous) based on FFQ	Total intracranial volume of (1) Grey matter (2) White matter	β (95% CI) (1) Grey matter: 0.01 (0.00, 0.01) (2) White matter: 0.001 (−0.005, 0.01)	Age, sex, education, vascular burden score, PASE, MIND*PASE	Fair
Zhang (2023) [30]	UK Biobank (UK)	Cross-sectional	N.A.	18214	Middle-aged to older adults (40-69y), free of dementia	14-MIND diet adherence (continuous) based on 2 to 4 Oxford webQs (web-based 24 h dietary assessment tool), scored according to quintiles of intake	Brain volume (mm^3^) measured by (1) Total brain (2) Grey matter (3) White matter (4) Superior frontal gyrus (5) Inferior frontal gyrus (6) Middle frontal gyrus (7) Supplementary motor cortex (8) Precentral gyrus (9) Postcentral gurus (10) Precuneus (11) Superior parietal lobe (12) Parahippocampal gyrus (13) Middle temporal gyrus (14) Inferior temporal gyrus (15) Hippocampus (16) Putamen (17) Thalamus (18) Caudate (19) Amygdala	β±SD (p-value, significance set at p < 6.6*10^−4^; multiple testing correction) (1) Total brain: 14.40 ± 469.36 (0.976) (2) Grey matter: −144.44 ± 276.68 (0.602) (3) White matter: 158.83 ± 304.02 (0.601) (4) Superior frontal gyrus: 2.11 ± 22.41 (0.925) (5) Inferior frontal gyrus: −13.71 ± 10.81 (0.205) (6) Middle frontal gyrus: −37.09 ± 22.86 (0.105) (7) Supplementary motor cortex: −12.74 ± 7.88 (0.106) (8) Precentral gyrus: −7.92 ± 22.45 (0.724) (9) Postcentral gurus: 21.24 ± 18.89 (0.261) (10) Precuneus: −6.28 ± 19.12 (0.743) (11) Superior parietal lobe: 22.46 ± 11.42 (0.049) (12) Parahippocampal gyrus: 13.60 ± 6.94 (0.050) (13) Middle temporal gyrus: 13.22 ± 19.88 (0.506) (14) Inferior temporal gyrus: 13.73 ± 16.33 (0.400) (15) Hippocampus: 12.40 ± 5.77 (0.032) (16) Putamen: 4.12 ± 6.52 (0.527) (17) Thalamus: 17.03 ± 9.73 (0.080) (18) Caudate: 7.53 ± 6.10 (0.217) (19) Amygdala: 4.77 ± 3.23 (0.140)	Age, sex, educational level, APOE, BMI, smoking status, alcohol consumption, regular physical activity, time on watching TV, sleep duration, Towsend deprivation index, family history of dementia, cancer, cardiovascular disease, diabetes	Fair
Chen (2021) [31]	Women's Health Initiative Hormone Replacement Therapy trial (USA)	Longitudinal	7–10	1302	Older woman (65-79y), free of dementia	15-MIND diet adherence (continuous, per 0.5 point) based on 122-item sFFQ	Brain volume (mm^3^) measured by (1) Total brain (2) Normal brain (excluding areas with evidence of small vessel ischemic disease) (3) Total white matter (4) Frontal lobe white matter (5) Parietal lobe white matter (6) Temporal lobe white matter (7) Corpus callosum white matter (8) Hippocampus	β (95% CI), adjusted p-value (1) Total brain: 0.10 (−0.17, 0.38), 0.90 (2) Normal brain: 0.23 (−0.15, 0.61), 0.90 (3) Total white matter: 0.74 (0.001, 1.48), 0.33 (4) Frontal lobe white matter: 0.33 (−0.01, 0.67), 0.33 (5) Parietal lobe white matter: 0.18 (−0.03, 0.39), 0.43 (6) Temporal lobe white matter: 0.19 (0.002, 0.37), 0.33 (7) Corpus callosum white matter: 0.001 (−0.02, 0.02), 0.90 (8) Hippocampus: 0.0007 (−0.02, 0.02), 0.90	Intracranial volume, age, race, U.S. regions, education level, employment, smoking status, alcohol consumption, BMI, physical activity, history of hypertension, diabetes, hypercholesterolemia, cardiovascular disease	Good

Abbreviations: AD: Alzheimer's disease, BMI: Body Mass index, CVD: Cardiovascular Disease, HDL: High-Density-Lipoprotein, MIND: Mediterranean-Dietary Approaches to Systolic Hypertension (DASH) diet Intervention for Neurodegenerative Delay, PASE: Physical Activity Scale for the Elderly, SD: Standard deviation, SE: Standard error, sFFQ: simplified Food Frequency Questionnaire. ^1^ Study quality was assessed with the Newcastle Ottawa Scale.

**TABLE 7:** Description of included randomized controlled trials describing the effect of the MIND diet on cognitive decline and brain volume

Author (year)	Study (country)	Duration (years)	Sample size (n)	Population	Exposure	Outcome	Results	Covariates	Study quality^1^
Arjmand (2022) [38]	MIND Diet Intervention and Cognitive Performance trial (Iran)	3 months	37	Obese middle-aged women (48 ± 5.38y), without any metabolic complication and free of dementia	14-MIND diet intervention with caloric restriction vs control diet with caloric restriction	Change in cognition measured by (1) Letter number sequencing task (LNST) (2) Auditory verbal learning test (AVLT) (3) Symbol digit modality task (SDMT) (4) Forward digit span task (FDST) (5) Backward digit span task (BDST) (6) Trail making test A (TMT A) (7) Trail making test B (TMT B) (8) Stroop task	Mean difference (95% CI) (1) LNST: 1.31 (0.79, 1.95), **p** ≤ **0.001** (2) AVLT: 1.54 (3.30, 6.40), **p** ≤ **0.001** (3) SDMT: 3.75 (2.43, 5.07), **p** ≤ **0.001** (4) FDST: 1.75 (1.15, 2.35), **p** ≤ **0.001** (5) BDST: 0.44 (0.01, 0.86), **p** = **0.041** (6) TMT A: −5.86 (−9.16, −2.22), **p** = **0.002** (7) TMT B: −2.63 (−6.34, 1.09), p = 0.161 (8) Stroop: −10.24 (−23.6, 3.09), p = 0.128 *(calculated based on given numbers)*	None	Some concerns
Barnes (2023) [55]	Trial of the MIND diet (USA)	3y	519-564 (n = 268–275 intervention group; depending on outcome)	Overweight older adults (≥65y), free of dementia	14-MIND diet intervention with mild caloric restriction vs control diet with mild caloric restriction	Change in cognition measured by (1) Global cognition (comp. of all tests below) (2) Episodic memory (comp. of word list memory, recall & recognition, East Boston story immediate & delayed recall) (3) Semantic memory (comp. of category fluency and multilingual naming test) (4) Executive functioning (comp. of TMT B and flanker inhibitory control and attention test) (5) Perceptual speed (comp. of oral symbol digit modality test, pattern comparison test, and TMT A)	Mean change between groups (95% CI) (1) Global cognition: 0.035 (−0.022, 0.092) (2) Episodic memory: 0.045 (−0.046, 0.137) (3) Semantic memory: −0.043 (−0.144, 0.057) (4) Executive functioning: 0.070 (−0.033, 0.173) (5) Perceptual speed: 0.008 (−0.078, 0.094)	None	Low bias
Barnes (2023) [55]	Trial of the MIND diet (USA)	3y	193-200 (97–101 intervention group; depending on outcome)	Overweight older adults (≥65y), free of dementia	14-MIND diet intervention with mild caloric restriction vs control diet with mild caloric restriction	Brain volume measured by (1) Grey and white matter (2) Hippocampal volume (3) White-matter hyperintense lesions	Mean change between groups (95% CI) (1) Grey and white matter: 0.001 (−0.003, 0.005) (2) Hippocampal: 0.005 (−0.016, 0.026) (3) White-matter hyperintense lesions: −0.019 (−0.046, 0.008)	Clinical site	Low bias

Abbreviations: AVLT: Auditory verbal learning test, BDST: Backward digit span task, CI: Confidence Interval, FDST: Forward digit span task, LNST: Letter number sequencing task, MIND: Mediterranean-Dietary Approaches to Systolic Hypertension (DASH) diet Intervention for Neurodegenerative Delay, SDMT: Symbol digit modality task, TMT: Trail making test. ^1^ Study quality was assessed with the Cochrane risk-of-bias tool for randomized trials.

The majority of included cohorts were conducted in North America (*n* = 12), followed by Europe (*n* = 11). The remaining studies were performed in Asia (*n* = 6), Australia (*n* = 2), and South America (*n* = 1).

In the articles with an observational design, MIND diet adherence was assessed as continuous measure, as quantiles, tertiles, and/or as low/high adherence. Adherence to the MIND diet was mostly assessed by food frequency questionnaires (FFQs); 5 cohorts used 24-h recalls [12,29,30,32,33], 2 cohorts used a short MIND adherence questionnaire [34,35], 1 cohort used a dietician interview [36], and 1 cohort used the combination of an FFQ and a 24-h recall [37]. In addition, interpretation and scoring of MIND diet components varied largely (Supplemental Table 9). Sample sizes ranged from *n* = 37 [38] to *n* = 114,684 [30]. The majority of included cohorts involved participants aged ≥60 y (*n* = 27) and participants free of dementia (*n* = 23).

### Cognitive function

A total of 14 articles with 13 unique cohorts assessed the cross-sectional association between adherence to the MIND diet and cognitive function. Cognitive function was either reported as global cognition composite (*n* = 5), domain-specific cognition (*n* = 7), or generic screening test outcome, such as Mini-Mental State Examination (MMSE) score or Telephone Interview for Cognitive Status (TICS) score (*n* = 5) (Table 1 [12,[26], [27], [28],^33^,^34^,^36^,^39^,[40], [41], [42], [43], [44], [45]]).

Among the 5 studies that assessed global cognition [[26], [27], [28],^39^,^40^], there were 4 unique cohorts, all originating from North America. Three of the 4 unique cohorts demonstrated a positive association between MIND adherence and global cognitive function. In 2 cohorts of middle-aged to older adults, a 1-point increase in MIND diet score was associated with β ± SE 0.03 ± 0.01 (*P* = 0.004) [28] and β = 0.027 (95% confidence interval [CI]: 0.008, 0.046) [40] point increase in global cognition (*z*-score). In addition, another cohort demonstrated that individuals in the lowest tertile of adherence to the MIND diet scored significantly worse on a global cognition composite compared to individuals with highest adherence (mean ± SE; T1 14.9 ± 0.10; T3 15.6 ± 0.09; *P*-trend < 0.001) [26]. The study by Berendsen et al. [39] was the only cohort that did not demonstrate an association. This cohort differed with respect to study population, as it was performed in female nurses rather than in an older general population of males and females. In addition, quality of this study was rated as fair, in contrast to the good quality of the other cohorts assessing global cognition.

Seven cohorts assessed domain-specific cognition [12,28,33,34,39,41,42], among which 3 were North American cohorts. Domain-specific cognition either involved composite scores that combined multiple tests into a domain [12,33,39,41,42] or single tests as a proxy for domain-specific cognition [28,34]. Episodic memory was positively associated with MIND diet adherence in 4 [12,28,39,41] out of 6 articles [33,42]. Higher MIND diet score was associated with better episodic memory composite (*z*-score) in Chinese (β_per 3 points_: 0.102; 95% CI: 0.051, 0.153) [12], German (β_per 1 point_: 0.045, 95% CI: 0.003, 0.087) [41], and North American (mean difference_Q1 compared with Q5_: 0.04, 95% CI: 0.01, 0.07) [39] cohorts. In addition, each point increase in MIND diet score was associated with improved visual reproductions delayed recall (β ± SE: 0.03 ± 0.01; *P* = 0.01) and logical memory delayed recall (β ± SE: 0.03 ± 0.01; *P* = 0.02) in another a North American cohort [28]. Two cohorts [33,42] did not find associations with episodic memory, though these studies had small sample sizes (*n* = 132 and *n* = 141, respectively). Evidence for the other cognitive domains is largely lacking. Positive associations were demonstrated for executive functioning in 2 [28,33] out of 5 cohorts [34,41,42], for processing speed in 1 [28] out of 2 cohorts [33], for working memory 1 [28] out of 4 cohorts [33,34,41], and for visuospatial memory 1 [28] out of 3 cohorts [34,41]. None of 2 cohorts found a beneficial association between better adherence to the MIND diet and semantic memory [41,42]. Among the 7 cohorts assessing domain-specific cognition, 4 cohorts were rated as good quality [12,28,33,41], 1 as fair [39], and 2 as poor [34,42]. The cohort rated as fair showed a positive association with episodic memory, and both cohorts with poor quality all showed null associations.

The generic tests were assessed in 6 cohorts [12,36,39,[43], [44], [45]]. Only 2 of these cohorts demonstrated a positive dose-response association between the level of adherence to the MIND diet and cognition [12,36]. A Greek cohort showed better MMSE performance in participants with better adherence to an adapted 9-point MIND score [*β* (*r*): 0.24 (0.32), *P* < 0.001; 95% CI/SD/SE not shown] [36]. In a Chinese cohort, higher adherence to a Chinese-adapted MIND diet was associated with better cognition as measured with the TICS-m (β: 0.110; 95% CI: 0.060, 0.159) [12]. Two studies also showed differences between tertiles of MIND adherence [43,44], but only the lowest and middle tertile of MIND diet adherence differed significantly rather than the lower and highest tertile. Finally, 2 cohorts did not find proof of an association between MIND diet adherence and cognition as measured with generic tests [39,45]. Overall, quality was low with only 2 articles scoring good [12,45], 2 fair [39,43], and 2 poor [36,44].

### Cognitive decline

Thirteen articles using data from 10 unique cohorts assessed the association between the adherence to the MIND diet and change in cognition. Change in cognition was reported as global cognition composite (*n* = 9), domain-specific cognition (*n* = 8), or a generic test score (*n* = 5) (Table 2 [4,12,20,22,24,28,35,39,40,[46], [47], [48], [49]]).

Of the 9 studies that studied global cognition [4,20,22,24,28,39,40,46,47], data from 7 unique cohorts were used. Five cohorts did not find associations between adherence to the MIND diet and change in global cognition [20,28,39,46,47], whereas 2 cohorts (presented in 5 articles) did demonstrate a positive association [4,20,22,24,40]. For each point increase in MIND diet score, global cognition increased with β = 0.0213 (95% CI: 0.008, 0.034) in a cohort of Puerto Ricans living in the United States [40] and with 0.0106 ± 0.0023 (β ± SE, *P* < 0.001) in the MAP cohort of older American adults [4]. The MIND diet was also protective of cognitive decline in a subpopulation of the MAP cohort with stroke [22]. Overall quality was good, with 7 articles scoring good [4,20,22,28,40,46,47], 1 scoring fair [39], and 1 scoring poor [24]. Of these lower-quality articles, 1 demonstrated a positive association [24], and 1 a null association [39].

With respect to change in domain-specific cognitive function, 7 unique cohorts were identified among the 8 articles that assessed this outcome [4,12,22,28,35,39,46,47]. Only the 2 articles using data from the American MAP cohort [4,22] and an Israeli study [47] demonstrated positive associations with change of domain-specific cognitive function in ≥1 domain. In the MAP cohort, Morris et al. [4] demonstrated that 1-point increase in MIND diet score was associated with an increase in episodic memory (β ± SE: 0.0090 ± 0.0028; *P* = 0.001), working memory (β ± SE: 0.0060 ± 0.0024; *P* = 0.01), semantic memory (β ± SE: 0.0113 ± 0.0027; *P* < 0.0001), visuospatial ability (β ± SE: 0.0077 ± 0.0025; *P* = 0.002), and perceptual speed (β ± SE: 0.0097 ± 0.0023; *P* < 0.0001). The Israeli study showed a positive association with each point increase in MIND diet score with executive functioning (β ± SE: 0.00978 ± 0.00446; *P* = 0.028), but not with episodic memory, attention, or language [47]. The other 5 cohorts, originating from North America, Europe, and Asia, did not show an association between adherence to the MIND diet and change in any cognitive domain [12,28,35,39,46]. The majority of articles were scored as good quality [4,22,28,46,47], with the exception of 3 articles [12,35,39]. These 3 studies all showed null associations.

Among the 5 studies that assessed change in cognition using generic tests [12,39,46,48,49], 2 demonstrated beneficial associations with better MIND adherence [48,49]. In 2 European cohorts of cognitively healthy older adults, MMSE increased by β = 0.006 (95% CI: 0.003, 0.009) per 1-point increase in MIND diet score [49] and STICS-m increased by β = 0.27 (95% CI: 0.05, 0.48) per 1.5 point increase in MIND diet score [48]. However, these 2 cohorts were rated as having poor [49] and fair [48] quality.

### Dementia

Eight articles using data from 10 unique cohorts studied the association between MIND diet adherence with risk of all-cause dementia and/or AD. In addition, 2 case–control studies assessed odds of dementia and early onset dementia (Table 3 [5,11,20,29,30,36,37,[50], [51], [52]]).

All-cause dementia was assessed in 7 articles including 10 cohorts [11,20,29,30,37,51,52], of which 7 out of 10 cohorts [11,20,37,51,52] showed that better adherence to the MIND diet was associated with a lower risk of all-cause dementia. Each point increase on a French-adapted MIND diet score was associated with a 10% lower risk of all-cause dementia (hazard ratio [HR]: 0.90; 95% CI: 0.83, 0.96) [37]. Positive associations were also observed in an Australian cohort (odds ratio [OR]: 0.72; 95% CI: 0.54, 0.95) [52], and 4 American cohorts (HR: 0.95; 95% CI: 0.92, 0.97; HR: 0.91; 95% CI: 0.83, 1.00; HR: 0.82, 95% CI: 0.68, 0.99; HR: 0.76, 95% CI: 0.57,1.00) [11,20]. Both positive and null associations were demonstrated in the same cohort from the Netherlands [51]; in one sample of participants better MIND diet adherence decreased risk of all-cause dementia over an average of 15.6 y (HR: 0.79; 95% CI: 0.70, 0.91), whereas another largely nonoverlapping sample that was followed for a mean of 5.9 y did not demonstrate an association (HR: 0.99; 95% CI: 0.94, 1.05). Finally, in 2 United Kingdom cohorts [11,29,30] and a biracial American cohort [20], no association with all-cause dementia was demonstrated. The majority of studies scored good on study quality [11,20,30,37,51] with 2 studies scoring fair [29,52]. The studies with fair quality demonstrated a positive association [52] and a null association [29].

Among the 3 studies that studied risk of AD [5,29,37], 2 showed beneficial associations [5,37]. The study of Morris et al. [5] showed the largest effect size: individuals in the American MAP cohort in the highest compared with lowest tertile of MIND diet adherence had 52% lower risk of developing AD (T1 compared with T3 HR: 0.48; 95% CI: 0.29, 0.79) [5]. These findings were confirmed in a sample of French older adults, with a French-adapted MIND diet score (HR: 0.89; 95% CI: 0.81, 0.97) [37]. Both studies scored good on quality. No association was demonstrated in a United Kingdom sample of older adults [29], which was rated as fair quality.

The 2 case–control studies on MIND adherence and dementia showed lower odds of dementia (OR: 0.43; 95% CI: 0.29, 0.63) [36], early onset dementia (OR: 0.66; 95% CI: 0.47, 0.91) and early onset AD (OR: 0.97; 95% CI: 0.46, 0.98) [50] but not for early onset frontotemporal dementia [50]. The study quality was rated as poor for both case–control studies.

### Cognitive impairment

An overview of all articles on cognitive impairment outcomes is shown in Table 4 [25,32,43,52,53].

Mild cognitive impairment (MCI) was assessed in 3 cohorts [43,52,53]. Two cohorts demonstrated protective associations: higher MIND diet adherence was cross-sectionally associated with lower odds of MCI in a Chinese a sample of older adults (T1 compared with T3 OR: 0.60; 95% CI: 0.51, 0.72) [43] and longitudinally with lower odds of MCI in Australian older adults after 12 y of follow-up (T1 compared with T3 OR: 0.47, 95% CI: 0.24, 0.91) [52]. The third cohort did not find a cross-sectional association between MIND diet adherence and odds of cognitive impairment in British PD patients (β: −0.23; 95% CI/SD/SE not shown; *P* = 0.070) [53]. The study quality was rated as fair [43], good [52], and poor [53].

The only study that assessed risk of subjective memory complaints was of good quality and demonstrated that better adherence to the MIND diet was associated with lower risk of memory complaints in older adults aged ≥70 y (HR: 0.87; 95% CI: 0.78, 0.98) but not in older adults aged 60–69 y (HR: 1.00; 95% CI: 0.95, 1.05) [32].

One study assessed the longitudinal association between cognitive resilience and adherence to the MIND diet. This study showed that higher MIND diet adherence was associated with higher cognitive resilience, based on change in global cognition adjusted for neuropathologies (mean difference: 0.07; 95% CI: 0.02, 012) [25]. The quality of the study was rated as good.

### PD

PD outcomes were assessed in 1 cross-sectional [54] and 1 longitudinal study [21] (Table 5). Cross-sectionally, Canadian PD patients adhering better to the MIND diet developed the disease at a later age (β: 2.2; 95% CI/SD/SE not shown; *P* = 0.002) [54]. Longitudinally, each point increase in MIND diet adherence was associated with a lower risk of incident PD (HR: 0.89; 95% CI: 0.83, 0.96) and a smaller change in PD progression (β ± SE: 0.008 ± 0.0037; *P* = 0.04) in the American MAP cohort [21]. The study quality of both studies was rated as poor.

### Brain volumes

Brain volume outcomes were assessed in 3 cross-sectional [28,30,42] and 1 longitudinal study [31] (Table 6). With respect to total brain volume, cross-sectional associations with MIND diet adherence were demonstrated in 1 (β_per 1 point_ ± SE: 0.02 ± 0.01; *P* = 0.02) [28] out of 2 cohorts [[30], [42]]. Longitudinally, MIND diet adherence was not associated with the change in total brain volume over 7–10 y [31]. Furthermore, no cross-sectional or longitudinal associations were demonstrated with grey matter (region), white matter (region), and subcortical areas [28,31,42]. Two studies were rated as good quality [28,31] and 2 as fair quality [30,42]. The studies with fair quality did not demonstrate any associations with brain volumes.

### Brain pathology

A total of 4 studies assessed neuropathologic markers, focusing on global AD pathology (*n* = 2), β-amyloid load (*n* = 2), tangles (*n* = 2), brain infarcts (*n* = 2), atherosclerosis (*n* = 1), and measures from cerebrospinal fluid (*n* = 1) [23,24,28,35] (Table 6).

Two studies made use of data of the American MAP cohort, resulting in 3 unique cohorts. Surprisingly, the 2 studies using data from the MAP study showed different results; although Agarwal et al. [23] demonstrated an association of MIND diet adherence with lower global AD pathology (β_continuous_ ± SE: −0.24 ± 0.011; *P* = 0.025) and β-amyloid load (β_T1 compared with T3_ ± SE: −0.246 ± 0.123; *P* = 0.047; β_continuous_ ± SE: −0.062 ± 0.034; *P* = 0.071) using an *n* = 581 sample from the MAP cohort, Dhana et al. [24] did not confirm this using data from *n* = 596 older individuals from the same cohort (global AD pathology: β_continuous_ ± SE: −0.013 ± 0.024; *P* = 0.578, β-amyloid: β_continuous_ ± SE: −0.03 ± 0.049; *P* = 0.395). Both MAP cohort studies did not demonstrate an association between MIND diet adherence and tau tangles. Furthermore, null associations between MIND diet adherence and brain infarcts [24,28], cerebral atherosclerosis [24], and cerebrospinal fluid biomarkers [35] were demonstrated. Quality was rated as good in 3 studies [23,24,28] and poor in 1 study [35]. The study of poor quality demonstrated a null association with cerebrospinal fluid biomarkers.

### RCTs

The effect of the MIND diet intervention on cognitive change and brain volume was reported in 2 articles (Table 7) [38,55]. In both articles, a calorie-restricted MIND diet was compared to a calorie-restricted control diet.

An American trial (*n* = 564) did not demonstrate an effect of a 3-y MIND diet intervention in older adults with overweight on change in global cognition (*z*-score) (mean change: 0.035; 95% CI: −0.022, 0.092), domain-specific cognition, and brain volumes [55]. This trial was rated as good quality, thus low risk of bias. A small Iranian trial (*n* = 37) in middle-aged females with obesity did demonstrate short-term beneficial effects of a MIND diet intervention. After a 3-mo intervention, the MIND diet group improved their cognitive functioning more compared to the control group on 6 of 8 cognitive tests, covering working memory, verbal memory, and attention domains. This article also included brain volume outcomes; as no effect sizes were reported, these data are not part of this systematic review. The study quality of the Iranian article was rated as with “some concerns of bias”.

## Discussion

In this review, we summarized the evidence on the MIND diet in relation to brain aging. The only intervention study with good quality did not demonstrate beneficial effects of a MIND diet intervention on cognition or brain volumes. With respect to observational research, the majority of studies indicated that the MIND diet reduces risk of all-cause dementia and AD. The evidence for the protective associations of the MIND diet with cognition, however, is more mixed. Although there are studies supporting cross-sectional associations with global cognition and episodic memory, these protective associations primarily originate from North American populations. In addition, longitudinal evidence as well as evidence for other cognitive domains is limited. Neuroimaging, pathology, and PD outcomes have only been addressed in few studies that so far do not hint toward benefits. Overall study quality was adequate, and excluding articles of poor or fair quality did not change the findings. Interestingly, the MIND diet works especially well for the MAP cohort, being the only cohort in which associations with brain pathology and cognitive decline in multiple domains has been demonstrated.

From a mechanistic point of view, protective associations could be expected as the MIND diet is rich in all nutrients considered relevant for healthy brain aging. Polyphenols and antioxidants from berries and vegetables and vitamin E from nuts and olive oil have anti-inflammatory, antioxidant, and/or vascular health-promoting properties [3]. ω-3 fatty acids from fish also possess these properties and act as building block for neurons [56]. Finally, B vitamins from leafy greens, whole grains, and poultry maintain homocysteine levels [57]. These multiple nutrients targeting different mechanisms are crucial, as the mechanisms underlying nutrition and brain aging are multifactorial [58]. This is further substantiated by the findings that evidence for dietary patterns is stronger than that for single nutrients and foods [3] and that nutrients have synergistic properties [59,60].

Our findings, however, do not conclusively prove the benefits of the MIND diet for brain aging. The only RCT with good quality did not show protective effects. Regarding observational studies, whereas we did find evidence for global cognitive functioning and dementia, the benefits of the MIND diet for global cognitive decline were only demonstrated in 2 out of 7 cohorts.

A possible explanation why the MIND diet trial showed null results is the choice of the control diet. In this trial, the effect of the MIND diet with mild caloric restriction was compared to a control diet with also mild caloric restriction. Over the 3 y of follow-up, both arms lost a similar amount of weight. Weight loss in itself may be responsible for improved cognition, that is, via lowering inflammation or improving insulin sensitivity, which may have overruled the benefits of the MIND diet intervention. Alternatively, selection bias could have occurred. The participants in the MIND diet trial were on average more highly educated and had a healthier medical history and higher baseline MIND diet score compared to participants of the MAP cohort in which the MIND diet was shown to be beneficial [4,5].

With respect to the observational evidence, a first hypothesis why the MIND diet works for some but not all cohorts is that the preferred diet for brain aging may be population-specific. This population dependency has already been demonstrated for Mediterranean and Nordic dietary patterns [49,61]. Better adherence to the Mediterranean diet was associated with a risk of all-cause mortality in both Mediterranean and non-Mediterranean countries, although effect sizes were larger in Mediterranean countries [61]. Similarly, in the context of brain aging, a Nordic dietary pattern was more strongly protectively associated with cognitive decline than the MIND diet in a Swedish population [49].

This hypothesis that the preferred diet for brain aging may be population-specific can be substantiated by differences in cultural practices between populations, which is an important factor influencing dietary behavior [62]. For example, a traditional Dutch way to consume leafy green vegetables is by eating “stamppot”, a dish that combines cooked leafy greens with mashed potatoes and meat. This is different from the way green leafy vegetables are likely being consumed in other countries, that is, raw as a salad. In addition, MIND diet-specific foods, such as berries, are not considered part of all cultures [63]. As a consequence, the MIND diet scoring system might capture different dietary patterns in different populations, depending on cultural practices.

The MIND diet may be the most preferred diet for brain aging in North America. This is supported by our findings, as cross-sectional protective associations were primarily observed in North American populations. The MIND diet was also especially protective for participants in the MAP cohort, the first cohort in which the MIND diet was tested [4,5]. Furthermore, some of the studies originating outside North America showing beneficial associations had adapted the MIND diet to their local eating habits. For example, a French study changed scoring thresholds to French guidelines and replaced berry intake by total polyphenol intake [37], and a Chinese study replaced wine by tea consumption [43]. Further research is required to discover if traditional eating habits with components of the MIND diet are more protective of brain aging than the original MIND diet.

Another possible explanation for the mixed findings is that study populations were not adequately selected. Preferably, there is a large variation in exposure and outcome between participants to allow easier detection of associations. In terms of exposure, this means a wide range of variation in dietary intake, that is, in MIND diet score. More variation in outcome can be achieved by selecting participants at risk of brain aging as opposed to the general population, as an at-risk population is more likely to decline. This can be exemplified by comparing the MAP cohort with the Nurses’ Health Study cohort, of which the MAP cohort did demonstrate beneficial associations [4], and the Nurses’ Health Study cohort did not [39]. Overall, there was more variation in MIND score in the MAP cohort compared to the Nurses’ Health Study cohort (2.5–12.5 compared with 2.6–11.0) and participants in the MAP cohort were at higher risk of cognitive decline compared to Nurses’ Health Study participants, as evidenced by a larger proportion of smokers and individuals with cardiovascular complaints [4,39].

Alternatively, it could be that focusing on diet only is a too simplistic view, as we know that many other factors can influence the association between the MIND diet and brain aging. For example, *APOE4* genotype may be an effect modifier, as reported by studies on other dietary patterns and brain aging [[64], [65], [66]]. Among our included studies, the interaction between *APOE4* genotype and the MIND diet has been demonstrated as well. Findings are inconsistent, however, with some studies reporting improved MIND diet-related brain aging among carriers [20,28,51], others among noncarriers [20], and the majority demonstrating no interaction [23,29,37,39,50].

In addition to genotype, other potential effect modifiers included income, physical activity, and exposure to fine particulate matter. Only individuals with higher income [67] or lower levels of physical activity [27,42] benefited from better adherence to the MIND diet. In addition, exposure to fine particulate matter was only harmful for brain aging in females not adhering well to the MIND diet [31]. These studies illustrate that the association between the MIND diet with brain aging is an interplay between many different factors.

The importance of interactions between various factors is now largely recognized and implemented in multidomain interventions. A well-known example is the FINGER (Finnish Geriatric Intervention Study to Prevent Cognitive Impairment and Disability) trial, the first RCT evidencing that a multidomain lifestyle intervention can slow cognitive decline in older adults at risk of dementia. Further building on this trial, the worldwide FINGERS network has been set up. This network of multidomain interventions for dementia prevention aims to extend the findings of FINGER to multiple populations and settings around the world. In several of these interventions, the MIND diet has been chosen as a basis for the nutrition component of the multidomain lifestyle (that is, US POINTER, clinicaltrials.gov NCT03688126; FINGER-NL, clinicaltrials.gov NCT05256199; LatAm-FINGERS [68]). These trials will give insight in the interplay between the MIND diet and other lifestyle factors in healthy brain aging.

Finally, our results should be interpreted with care because of several methodologic limitations. There was a large variation in exposure assessment, with differences in dietary assessment methods (FFQ, food dairy), timing of assessment, and interpretation and scoring of MIND components that limits comparability between studies. In addition, measurement of outcomes varied largely. Without consensus on the optimal neuropsychologic test battery to capture cognitive changes, especially in the preclinical phase, and no rules on how to construct cognitive domains, it is hard to draw firm conclusions [69]. Because of this heterogeneity in outcomes, we chose to not perform a meta-analysis. Also, as the majority of included studies had an observational design, there is a risk of reverse causation, residual confounding, and over-adjustment. Another limitation is that many articles made use of data from the MAP cohort, which may give a limited perspective on the state of evidence. Finally, we assessed quality of individual articles using NOS and ROB2, but we did not assess overall quality of evidence using, for example, the GRADE approach.

To conclude, this systematic review shows observational evidence for a beneficial association between the MIND diet with global cognitive function and dementia risk, but evidence for cognitive decline, cognitive impairment, brain volume, pathology, and PD remains mixed and/or limited. The preferred diet for brain aging may be population-specific, with the MIND diet being the favored diet for North American populations.

## Author contributions

The authors’ contributions were as follows – AvS, SB, OvdR, LdG: designed the research; AvS, SB: performed the systematic literature search, screened publications for eligibility, extracted the data and scored the study quality, and wrote the manuscript; and all authors: interpreted the results, had responsibility for the final content, and read and approved the final manuscript.

## Funding

The authors reported no funding received for this study.

## Conflict of interest

Lisette de Groot is an Editor for Advances in Nutrition and played no role in the Journal’s evaluation of the manuscript. All other authors report no conflicts of interest.
