# Supplementary material for: The Mediterranean-Dietary Approaches to Stop Hypertension Intervention for Neurodegenerative Delay (MIND) Diet for the Aging Brain: A Systematic Review
Source: Adv Nutr. 2024 Feb 3;15(3):100184. doi: 10.1016/j.advnut.2024.100184 (PMC10942868; doi:10.1016/j.advnut.2024.100184)
Supplement: Multimedia component 1 [file mmc1.docx]

**Supplementary Materials**

**Table S1A**: Search strategy Ovid Medline

|  | ***Ovid Medline*: 12-10-2022** | **Hits** |
| --- | --- | --- |
| **1** | MIND diet*.mp | 105 |
| **2** | Mediterranean-DASH.mp | 97 |
| **3** | 1 or 2 | 134 |
| **4** | cognit*.mp | 545719 |
| **5** | Exp Dementia/ | 195928 |
| **6** | dementia.mp | 151270 |
| **7** | Alzheimer*.mp | 190802 |
| **8** | parkinson*.mp | 150303 |
| **9** | brain.mp | 1566911 |
| **10** | 4 or 5 or 6 or 7 or 8 or 9 | 2186540 |
| **11** | 3 and 10 | 91 |

**Table S1B**: Search strategy Web of Science core collection

|  | ***Web of Science core collection*:   12-10-2022** | **Hits** |
| --- | --- | --- |
| **1** | ALL=(“MIND diet*”) | 112 |
| **2** | ALL=(“Mediterranean-DASH”) | 88 |
| **3** | #1 or #2 | 136 |
| **4** | ALL=(cognit*) | 984686 |
| **5** | ALL=(dementia) | 207765 |
| **6** | ALL=(Alzheimer*) | 298393 |
| **7** | ALL=(Parkinson*) | 233128 |
| **8** | ALL=(brain) | 1865420 |
| **9** | 4 or 5 or 6 or 7 or 8 | 2842640 |
| **10** | #3 and #9 | 108 |

**Table S1C**: Search strategy Scopus

|  | ***Scopus*: 12-10-2022** | **Hits** |
| --- | --- | --- |
| **1** | TITLE-ABS-KEY(“MIND diet*”) | 143 |
| **2** | TITLE-ABS-KEY({Mediterranean-DASH}) | 84 |
| **3** | #1 OR #2 | 157 |
| **4** | TITLE-ABS-KEY(cognit*) | 1080735 |
| **5** | TITLE-ABS-KEY (dementia) | 221084 |
| **6** | TITLE-ABS-KEY(alzheimer*) | 259343 |
| **7** | TITLE-ABS-KEY(parkinson*) | 209188 |
| **8** | TITLE-ABS-KEY(brain) | 2437047 |
| **9** | 4 OR 5 OR 6 OR 7 OR 8 | 3535872 |
| **10** | #3 AND #9 | 111 |

**Table S2.** NEWCASTLE - OTTAWA QUALITY ASSESSMENT SCALE (NOS) COHORT STUDIES

| Selection (max. 4) |
| --- |
| 1) Representativeness of the exposed cohort  a) truly representative of the average in target population in the community *  b) somewhat representative of the average in target population in the community *  c) selected group of users e.g. nurses, volunteers  d) no description of the derivation of the cohort |
| 2) Selection of the non-exposed cohort  a) drawn from the same community as the exposed cohort *  b) drawn from a different source  c) no description of the derivation of the non-exposed cohort |
| 3) Ascertainment of exposure (MIND diet)  a) FFQ/ ≥3x 24h recall/ food diary AND MIND total score ≥13 points *  b) structured (dietician) interview AND MIND total score ≥13 points *  c) written self-report OR MIND total score <13 points  d) no description |
| 4) Demonstration that outcome of interest was not present at start of study  a) yes *  b) no |
| Comparability (max 2) |
| 1) Comparability of cohorts on the basis of the design or analysis  a) study controls for age, sex, and education *  b) study controls for any additional lifestyle or genetic factor (e.g. smoking, alcohol, physical activity, BMI, APOE4) * |
| Outcome (max 4) |
| 1) Assessment of outcome  a) independent or blind assessment *  b) record linkage *  c) self-report  d) no description |
| 2) Was follow-up long enough for outcomes to occur  a) yes (≥2 years for cognitive decline, MRI data and brain pathology, ≥5 years for dementia/MCI incidence and cognitive screeners (e.g. MMSE, MoCA, TICs ) *  b) no |
| 3) Adequacy of follow up of cohorts  a) complete follow up - all subjects accounted for *  b) subjects lost to follow up unlikely to introduce bias - small number lost - > 70% follow up, or description provided of those lost *  c) follow up rate < 70% and no description of those lost  d) no statement |
| 4) Statistical test  a. The statistical test used to analyse the data is clearly described and appropriate, and the measurement of the association is presented including confidence intervals and the probability level (p value) * b. The statistical test is not appropriate, not described or incomplete |

Note: A study can be awarded a maximum of one star for each numbered item within the Selection and Outcome categories. A maximum of two stars can be given for the comparability question.

Scoring: Good quality: ≥3 stars in selection domain AND 2 stars in comparability domain AND ≥2 stars in outcome domain. Fair quality: ≥2 stars in selection domain AND ≥1 stars in comparability domain AND ≥2 stars in outcome domain. Poor quality: <2 stars in selection domain OR 0 stars in comparability domain OR <2 stars in outcome domain.

**Table S3.** NEWCASTLE - OTTAWA QUALITY ASSESSMENT SCALE (NOS) CASE CONTROL STUDIES

| Selection (max. 4) |
| --- |
| 1) Is the case definition adequate?  a) yes, with independent validation (e.g. >1 person/record/time/process to extract information, or reference to   primary record source such as x-rays or medical/hospital records)*  b) yes, eg record linkage*  c) based on self-reports  d) no description |
| 2) Representativeness of the cases  a) consecutive or obviously representative series of cases (All eligible cases with outcome of interest over a defined  period of time, all cases in a defined catchment area, all cases in a defined hospital or clinic, group of hospitals, health  maintenance organisation, or an appropriate sample of those cases (e.g. random sample) *  b) potential for selection biases or not stated |
| 3) Selection of Controls  a) community controls (same community as cases) *  b) hospital controls (within same community as cases, but derived from hospitalised population)  c) no description |
| 4) Definition of Controls  a) no history of disease (endpoint) *  b) no description of source |
| Comparability (max 2) |
| 1) Comparability of cases and controls on the basis of the design or analysis  a) study controls for age, sex, and education *  b) study controls for any additional lifestyle or genetic factor (e.g. smoking, alcohol, physical activity, BMI, APOE4) * |
| Exposure (max 4) |
| 1) Ascertainment of exposure (MIND diet)  a) FFQ/ ≥3x 24h recall/ food diary AND MIND total score ≥13 points *  b) structured (dietician) interview AND MIND total score ≥13 points *  c) written self-report OR MIND total score <13 points  d) no description |
| 2) Same method of ascertainment for cases and controls  a) yes *  b) no |
| 3) Non-Response rate (drop-outs)  a) same rate for both groups *  b) non respondents described  c) rate different and no designation |
| 4) Statistical test  a. The statistical test used to analyse the data is clearly described and appropriate, and the measurement of the association is presented including confidence intervals and the probability level (p value) * b. The statistical test is not appropriate, not described or incomplete |

Note: A study can be awarded a maximum of one star for each numbered item within the Selection and Outcome categories. A maximum of two stars can be given for the comparability question.

Scoring: Good quality: ≥3 stars in selection domain AND 2 stars in comparability domain AND ≥3 stars in exposure domain. Fair quality: ≥2 stars in selection domain AND ≥1 stars in comparability domain AND ≥3 stars in exposure domain. Poor quality: <2 stars in selection domain OR 0 stars in comparability domain OR <3 stars in exposure domain.

**Table S4.** NEWCASTLE - OTTAWA QUALITY ASSESSMENT SCALE (NOS) ADOPTED FOR CROSS-SECTIONAL STUDIES

|  | Selection (max. 3) |
| --- | --- |
|  | Representative of the sample a. Truly representative of the average in target population in the target population (random sample or whole population) ***** b. Somewhat representative of the average in target population in the target population (non-random sample) ***** c. Selected group/convenience sample d. No description of the sampling strategy |
|  | Non-respondents a. Comparability between respondents and non-respondents characteristics is established, **or** the response rate is satisfactory (>70%)* b. The response rate is unsatisfactory, **and** the comparability between respondents and non-respondents is unsatisfactory c. No description of the response rate or the characteristics of the responders and non-responders |
|  | Ascertainment of the exposure (MIND diet)  a) FFQ/ ≥3x 24h recall/ food diary AND MIND total score ≥13 points *****  b) structured (dietician) interview AND MIND total score ≥13 points *****  c) written self-report OR MIND total score <13 points  d) no description |
|  | **Comparability (max. 2)** |
|  | The subjects in different outcome groups are comparable, based on the study design or analysis. Confounding factors are controlled for a. Study controls for age, sex, and education *****  b. study controls for any additional lifestyle or genetic factor (e.g. smoking, alcohol, physical activity, BMI, APOE4) ***** |
|  | **Outcome (max. 2)** |
|  | Assessment of the outcome (brain health) a. independent or blind assessment ***** b. record linkage ***** c. Self-report d. no description |
|  | Statistical test a. The statistical test used to analyse the data is clearly described and appropriate, and the measurement of the association is presented including confidence intervals and the probability level (p value) ***** b. The statistical test is not appropriate, not described or incomplete |

Note: This scale was a modified version of the NOS scale, as used in several other review studies.
A maximum of two point can be given for Comparability. Scoring: Good quality: ≥2 stars in selection domain AND ≥2 stars in comparability domain AND ≥1 stars in outcome domain. Fair quality: ≥1 stars in selection domain AND ≥1 stars in comparability domain AND ≥1 stars in outcome domain. Poor quality: 0 stars in selection domain OR 0 stars in comparability domain OR 0 stars in outcome domain.

**Table S5.** The Newcastle-Ottawa Scale (NOS) scores for cohort studies included in the review

| Study (authors) | Outcome category | Representative of exposed sample | Selection non-exposed | Ascertainment exposure | Not present at start | Design or analysis | Assessment of outcome | Follow-up | Adequacy follow-up | Statistics | Study Quality |
| --- | --- | --- | --- | --- | --- | --- | --- | --- | --- | --- | --- |
| Van Lent (2021) | Cognitive decline | 1 | 1 | 1 | 0 | 2 | 0 | 1 | 1 | 1 | Good |
| Dhana (2021) | Cognitive decline | 1 | 1 | 1 | 0 | 1 | 0 | 0 | 0 | 1 | Poor |
| Cherian (2019) | Cognitive decline | 1 | 1 | 1 | 0 | 2 | 1 | 1 | 0 | 1 | Good |
| Morris (2015) | Cognitive decline | 1 | 1 | 1 | 0 | 2 | 1 | 1 | 1 | 1 | Good |
| Berendsen (2018) | Cognitive decline | 0 | 1 | 1 | 0 | 2 | 1 | 1 | 1 | 1 | Fair |
| Nishi (2021) | Cognitive decline | 1 | 1 | 1 | 1 | 2 | 0 | 1 | 1 | 1 | Good |
| Vu (2022) - CHAP | Cognitive decline | 1 | 1 | 1 | 0 | 2 | 1 | 0 | 0 | 1 | Good |
| Vu (2022) - MAP | Cognitive decline | 1 | 1 | 1 | 0 | 2 | 1 | 0 | 0 | 1 | Good |
| Boumenna (2022) | Cognitive decline | 1 | 1 | 1 | 0 | 2 | 1 | 1 | 1 | 1 | Good |
| Munoz-Garcia (2020) | Cognitive decline | 0 | 1 | 1 | 0 | 2 | 0 | 1 | 1 | 1 | Fair |
| Shakersain (2018) | Cognitive decline | 0 | 1 | 0 | 0 | 2 | 0 | 1 | 0 | 1 | Poor |
| Lotan (2022) | Cognitive decline | 1 | 1 | 1 | 0 | 2 | 1 | 1 | 1 | 1 | Good |
| Huang (2023) | Cognitive decline | 1 | 1 | 0 | 0 | 2 | 0 | 0 | 1 | 1 | Fair |
| Adjibade (2019) | Subjective memory complaints | 1 | 1 | 1 | 1 | 2 | 1 | 1 | 1 | 1 | Good |
| Dong (2023) | Cognitive decline | 1 | 1 | 0 | 0 | 0 | 0 | 0 | 0 | 0 | Poor |
| Hosking (2019 | Mild cognitive impairment | 1 | 1 | 1 | 1 | 2 | 1 | 1 | 1 | 1 | Good |
| Thomas (2022) | Dementia | 1 | 1 | 1 | 1 | 2 | 1 | 1 | 0 | 1 | Good |
| Morris (2015) | Dementia | 1 | 1 | 1 | 1 | 2 | 1 | 0 | 0 | 1 | Good |
| Vu (2022) - CHAP | Dementia | 1 | 1 | 1 | 1 | 2 | 1 | 0 | 0 | 1 | Good |
| Vu (2022) - MAP | Dementia | 1 | 1 | 1 | 1 | 2 | 1 | 0 | 0 | 1 | Good |
| Vu (2022) - WHIMS | Dementia | 1 | 1 | 1 | 1 | 2 | 1 | 0 | 1 | 1 | Good |
| de Crom (2022) | Dementia | 1 | 1 | 1 | 1 | 2 | 1 | 1 | 0 | 1 | Good |
| Hosking (2019) | Dementia | 1 | 1 | 1 | 1 | 1 | 1 | 1 | 1 | 1 | Fair |
| Cornelis (2023) | Dementia | 1 | 1 | 0 | 0 | 2 | 1 | 1 | 1 | 1 | Fair |
| Zhang (2023) | Dementia | 1 | 1 | 0 | 1 | 2 | 0 | 1 | 0 | 1 | Good |
| Chen (2023), Whitehall II study | Dementia | 0 | 1 | 1 | 1 | 2 | 1 | 1 | 1 | 1 | Good |
| Chen (2023), Health and Retirement Study | Dementia | 1 | 1 | 1 | 1 | 2 | 1 | 0 | 1 | 1 | Good |
| Chen (2023), Framingham Heart Study | Dementia | 1 | 1 | 1 | 1 | 2 | 1 | 1 | 0 | 1 | Good |
| Agarwal (2018) | Parkinson's disease | 1 | 1 | 1 | 1 | 1 | 1 | 0 | 0 | 0 | Poor |
| Dhana (2021) | Brain pathology | 1 | 1 | 1 | 0 | 2 | 1 | 0 | 0 | 1 | Good |
| Chen (2021) | Brain volumes | 1 | 1 | 1 | 0 | 2 | 1 | 1 | 1 | 1 | Good |
| Agarwal (2023) | Brain pathology | 1 | 1 | 1 | 0 | 2 | 1 | 1 | 1 | 1 | Good |
| Dong (2023) | Brain pathology | 1 | 1 | 0 | 0 | 0 | 1 | 0 | 0 | 0 | Poor |
| Wagner (2023) | Cognitive resilience | 1 | 1 | 1 | 0 | 2 | 1 | 1 | 0 | 1 | Good |

**Table S6.** The Newcastle-Ottawa Scale (NOS) scores for case-control studies included in the review

| Author (year) | Outcome category | Case definition adequate | Representativeness cases | Selection controls | Definition controls | Design or analysis | Assessement of exposure | Same method | Non-response rate | Statistics | Study Quality |
| --- | --- | --- | --- | --- | --- | --- | --- | --- | --- | --- | --- |
| Vassilopoulou (2022) | Dementia | 1 | 0 | 0 | 1 | 1 | 0 | 1 | 0 | 1 | Poor |
| Filippini (2020) | Dementia | 0 | 0 | 1 | 0 | 2 | 1 | 1 | 1 | 1 | Poor |

**Table S7.** The Newcastle-Ottawa Scale (NOS) scores for cross-sectional studies included in the review

| Author (year) | Outcome category | Representative of exposed sample | Non-respondents | Ascertainment exposure | Design or analysis | Assessment of outcome | Statistics | Study Quality |
| --- | --- | --- | --- | --- | --- | --- | --- | --- |
| Van Lent (2021) | Cognitive function | 1 | 1 | 1 | 2 | 0 | 1 | Good |
| Vassilopoulou (2022) | Cognitive function | 0 | 0 | 0 | 1 | 1 | 0 | Poor |
| Berendsen (2018) | Cognitive function | 0 | 0 | 1 | 2 | 1 | 1 | Fair |
| Calil (2018) | Cognitive function | 0 | 0 | 1 | 0 | 1 | 1 | Poor |
| McEvoy (2017) | Cognitive function | 1 | 1 | 1 | 2 | 0 | 1 | Good |
| Gauci (2022) | Cognitive function | 1 | 1 | 0 | 2 | 1 | 0 | Good |
| Huang (2022) | Cognitive function | 1 | 0 | 0 | 2 | 0 | 1 | Fair |
| Ahn (2022) | Cognitive function | 1 | 0 | 1 | 2 | 0 | 1 | Good |
| Boumenna (2022) | Cognitive function | 1 | 1 | 1 | 2 | 1 | 1 | Good |
| Yeung (2022) | Cognitive function | 1 | 1 | 0 | 2 | 0 | 1 | Good |
| Wesselman (2021) | Cognitive function | 1 | 1 | 1 | 2 | 0 | 1 | Good |
| Escher (2022) | Cognitive function | 0 | 0 | 1 | 2 | 0 | 0 | Poor |
| Huang (2023) | Cognitive function | 1 | 1 | 0 | 2 | 0 | 1 | Good |
| Zare (2023) | Cognitive function | 1 | 0 | 0 | 0 | 1 | 0 | Poor |
| Lawrie (2022) | Mild Cognitive Impairment | 1 | 0 | 1 | 2 | 0 | 0 | Poor |
| Huang (2022) | Mild Cognitive Impairment | 1 | 0 | 0 | 2 | 0 | 1 | Fair |
| Metcalfe-Roach (2021) | Parkinson's disease | 1 | 0 | 1 | 2 | 0 | 0 | Poor |
| van Lent (2021) | Brain Volumes | 1 | 0 | 1 | 2 | 1 | 1 | Good |
| Escher (2022) | Brain Volumes | 0 | 0 | 1 | 2 | 1 | 1 | Fair |
| Zhang (2023) | Brain Volumes | 1 | 1 | 1 | 2 | 1 | 1 | Fair |

**Table S8. The Cochrane Risk of Bias tool in Randomized Controlled Trials (Rob2) scoring of studies included in the review**

| Study (authors) | Outcome category | Randomization Process | Deviation from intended interventions | Missing outcome data | Measurement of the outcome | Selection of the reported result | Overall Bias |
| --- | --- | --- | --- | --- | --- | --- | --- |
| Arjmand (2022) | Cognition | 0 | + | + | + | 0 | 0 |
| Barnes (2023) | Cognition | + | + | + | + | + | + |
| Barnes (2023) | Brain Volumes | + | + | + | + | + | + |

Key: +: Low risk of bias; 0: some concerns; -: high concerns; n/a: not assessed.

**Table S9. Overview MIND diet scoring methodology per included article.**

| Author (year) | Components | | | Scoring | | |
| --- | --- | --- | --- | --- | --- | --- |
|  | **# of items** | **Similar to original MIND diet** | **Explanation** | **Type of scoring** | **Similar to original MIND diet** | **Explanation** |
| McEvoy (2017) | 15 | No | Several cabbages considered green leafy vegetable; peas considered beans | Based on set serving size | Yes | n/a |
| Ahn (2022) | 15 | Unknown | No detailed food classification provided | Based on set serving size | Unknown | Referred to original article, but no additional explanation given |
| Van Lent (2021) | 15 | Yes | n/a | Based on set serving size | Yes | n/a |
| Berendsen (2018) | 15 | Unknown | No detailed food classification provided | Based on set serving size | Yes | n/a |
| Boumenna (2022) | 15 | Unknown | No detailed food classification provided | Based on set serving size | Unknown | Referred to original article, but no additional explanation given |
| Huang (2023) | 12 | No | Excluded: olive oil, butter/margarine, and cheese. Red wine replaced with tea | Based on cut-points | No | Based on tertiles |
| Wesselman (2021) | 15 | Yes | n/a | Based on set serving size | Yes | n/a |
| Escher (2022) | 15 | Unknown | No detailed food classification provided | Based on set serving size | Yes | n/a |
| Gauci (2022) | 15 | Unknown | No detailed food classification provided | Based on set serving size | Yes | n/a |
| Zare (2023) | 14 | No | Excluded: wine | Based on set serving size | Yes | n/a |
| Huang (2022) | 12 | No | Replaced: whole grains to type of staple food, berries to fresh fruit, beans to soybeans, olive oil to vegetable oil, wine to tea, green leafy vegetable and other vegetables to fresh vegetables and mushroom/ algae. Removed: Poultry, butter/ margarine, cheese, red meat and products, fast fried foods. Added: Garlic | Based on set serving size | No | Scoring differs for all components but fish and nuts |
| Vassilopoulou (2022) | 9 | No | Excluded: other vegetables, beans, poultry, cheese, olive oil, red meat | Based on set serving size | Unknown | Referred to original article, but additional explanation inadequate to make a proper comparison |
| Calil (2018) | 15 | Yes | n/a | Based on set serving size | No | Scoring differs for vegetables and whole cereals |
| Yeung (2022) | 9 | No | Excluded: olive oil, beans, fish, poultry, fried/fast foods, and red meat and products | Based on set serving size | Unknown | Not reported |
| Vu (2022) | 15 | Unknown | Supplementary table cannot be opened | Based on set serving size | Unknown | Supplementary table cannot be opened |
| Cherian (2019) | 15 | Unknown | No detailed food classification provided | Based on set serving size | Unknown | Referred to original article, but no additional explanation given |
| Morris (2015a) | 15 | Yes | Original article | Based on set serving size | Yes | Original article |
| Dhana (2021) | 15 | Unknown | No detailed food classification provided | Based on set serving size | Unknown | Not reported |
| Nishi (2021) | 15 | Unknown | No detailed food classification provided | Based on set serving size | Unknown | Referred to original article, but no additional explanation given |
| Lotan (2022) | 15 | Unknown | No detailed food classification provided | Based on set serving size | Unknown | Referred to original article, but no additional explanation given |
| Dong (2023) | 15 | Unknown | No detailed food classification provided | Based on set serving size | Unknown | Referred to original article, but no additional explanation given |
| Munoz-Garcia (2020) | 15 | Unknown | No detailed food classification provided | Based on set serving size | Yes | n/a |
| Shakersain (2018) | 14 | No | Replaced: olive oil to vegetable oil. Excluded: nuts | Based on cut-points | No | Based on sex-specific population median. For brain healthy foods, intake below the median was scored 0 and scores 1 to 5 were assigned to quintiles of intakes above the median. For brain unhealthy foods, scoring was reversed. |
| Filippini (2020) | 15 | Unknown | No detailed food classification provided | Unknown | Unknown | Referred to original article, but no additional explanation given |
| Thomas (2022) | 15 | No | Replaced: berry intake to total polyphenol intake | Based on set serving size | No | Scoring adapted to French dietary habits and guidelines for fish, other vegetables, green leafy vegetables, nuts, whole grain |
| Morris (2015b) | 15 | Yes | Original article | Based on set serving size | Yes | Original article |
| de Crom (2022) | 15 | No | Cabbage included as green leafy vegetable, flax seeds included as nuts, mussels included as fish | Unknown | Unknown | Referred to original article, but no additional explanation given |
| Hosking (2019) | 13 | No | Excluded: butter/margarine and olive oil | Unknown | Unknown | Referred to original article, but no additional explanation given |
| Cornelis (2023) | 15 | No | Seeds included as nuts, shellfish included as fish | Based on set serving size | No | For butter/margarine scoring is reported to be opposite to original (might be error in reporting). Scoring differs for wine |
| Zhang (2023) | 14 | No | Excluded: olive oil | Based on cut-points | No | Based on quintiles |
| Chen (2023) - WII cohort | 14 | No | Excluded: olive oil | Based on set serving size | No | Scoring differs for berries, nuts and whole grains (whole grains might be error in reporting) |
| Chen (2023) - HRS & FHS - offspring | 15 | Unknown | No detailed food classification provided | Based on set serving size | No | Scoring differs for berries, nuts and whole grains (whole grains might be error in reporting) |
| Lawrie (2022) | 15 | Unknown | Supplementary table cannot be opened | Based on set serving size | Unknown | Referred to original article, but no additional explanation given |
| Adjibade (2019) | 15 | Unknown | No detailed food classification provided | Based on set serving size | Yes | n/a |
| Wagner (2023) | 15 | Unknown | No detailed food classification provided | Based on set serving size | Unknown | Referred to original article, but no additional explanation given |
| Metcalfe-Roach (2021) | 15 | Unknown | No detailed food classification provided | Based on set serving size | Yes | n/a |
| Agarwal (2018) | 15 | Unknown | No detailed food classification provided | Based on set serving size | Unknown | Referred to original article, but no additional explanation given |
| Agarwal (2023) | 15 | Yes | n/a | Based on serving size | Unknown | Referred to original article, but no additional explanation given |
| Chen (2021) | 15 | No | Canola included as olive oil. Kiwi included as berry. Seeds included as nuts. | Based on serving size | No | Scoring differs for whole grains, butter/margarine |
| Arjmand (2022) | 15 | No | Replaced: wine to grape juice | Based on serving size | Yes | n/a |
| Barnes (2023) | 14 | No | Excluded: wine | Unknown | Unknown | Not reported |
